# Lifetime Health Effects and Cost-Effectiveness of Tirzepatide and Semaglutide in US Adults

**DOI:** 10.1001/jamahealthforum.2024.5586

**Published:** 2025-03-14

**Authors:** Jennifer H. Hwang, Neda Laiteerapong, Elbert S. Huang, David D. Kim

**Affiliations:** 1 Section of General Internal Medicine, Department of Medicine, University of Chicago, Chicago, Illinois; 2 Department of Psychiatry and Behavioral Neuroscience, University of Chicago, Chicago, Illinois; 3 Department of Medicine, University of Chicago, Chicago, Illinois; 4 Department of Public Health Sciences, University of Chicago, Chicago, Illinois

## Abstract

**Importance:**

Newer antiobesity medications lead to greater weight loss and lower cardiometabolic risks. However, the high costs of these medications have raised policy questions about their value and coverage decisions.

**Objective:**

To compare the cost-effectiveness of 4 antiobesity medications with lifestyle modification vs lifestyle modification alone in the US.

**Design, Setting, and Participants:**

A lifetime cost-effectiveness analysis was conducted in 2024 using the validated Diabetes, Obesity, Cardiovascular Disease Microsimulation model for US adults. Data were included from the 2017-2020 National Health and Nutrition Examination Survey of 4823 individuals (representing 126 million eligible US adults) aged 20 to 79 years who would meet clinical trial inclusion criteria for antiobesity medications. Individual-level simulations projected long-term cardiometabolic outcomes, quality-adjusted life-years (QALYs), and health care expenditures. Probabilistic sensitivity analyses, subgroup analyses (across body mass index [BMI] categories [≥30 or ≥27 and at least 1 weight-related comorbidity], presence of comorbidities), and multiple scenario analyses (varying treatment discontinuation rates, value-based pricing benchmarks) were conducted. Future costs and QALYs were discounted at 3% annually.

**Interventions:**

Lifestyle modification with naltrexone-bupropion, phentermine-topiramate, semaglutide, or tirzepatide vs lifestyle modification alone.

**Main Outcomes and Measures:**

Obesity, diabetes, and cardiovascular disease cases averted, life-years and QALYs gained, costs incurred (2023 US dollars), and incremental cost-effectiveness ratios.

**Results:**

Among the 126 million eligible US adults, the mean age was 48 (SE, 0.5) years; 51% were female; and the initial mean BMI was 34.7 (SE, 0.2); and 85% had at least 1 weight-related comorbidity. Over a lifetime, tirzepatide would avert 45 609 obesity cases (95% uncertainty interval [UI], 45 092-46 126) per 100 000 individuals and semaglutide would avert 32 087 cases (95% UI, 31 292-32 882) per 100 000 individuals. Tirzepatide would reduce 20 854 incident cases of diabetes (95% UI, 19 432-22 276) per 100 000 individuals and semaglutide would reduce 19 211 cases (95% UI, 17 878-20 544) per 100 000 individuals. Tirzepatide would reduce 10 655 cardiovascular disease cases (95% UI, 10 124-11 186) per 100 000 individuals and semaglutide would reduce 8263 cases (95% UI, 7738-8788) per 100 000 individuals. Despite the largest incremental QALY gains of 0.35 for tirzepatide and 0.25 for semaglutide among all antiobesity medications, the incremental cost-effectiveness ratios were $197 023/QALY and 467 676/QALY, respectively. To reach the $100 000/QALY threshold, their prices would require additional discounts by 30.5% for tirzepatide and 81.9% for semaglutide from their current net prices. Naltrexone-bupropion was cost saving due to its lower cost and had an 89.1% probability of being cost-effective at $100 000/QALY, whereas phentermine-topiramate had a 23.5% probability of being cost-effective at $100 000/QALY. Tirzepatide and semaglutide both had a 0% probability across all QALY threshold ranges examined ($100 000-$200 000/QALY).

**Conclusions and Relevance:**

This economic evaluation found that although tirzepatide and semaglutide offered substantial long-term health benefits, they were not cost-effective at current net prices. Efforts to reduce the net prices of new antiobesity medications are essential to ensure equitable access to highly effective antiobesity medications.

## Key Points

**Question:**

What are the lifetime health effects and cost-effectiveness of different antiobesity medications compared with lifestyle modification in the US population?

**Findings:**

In this economic evaluation of 4 antiobesity medications, tirzepatide and semaglutide were found to generate greater lifetime health gains by preventing diabetes, cardiovascular complications, and death compared with phentermine-topiramate and naltrexone-bupropion; however, they are not cost-effective at their current net prices.

**Meaning:**

These results support the need for ongoing efforts to further reduce the net prices of new antiobesity medications to ensure equitable access to these highly effective medications.

## Teaser

This study compares the cost-effectiveness of 4 antiobesity medications (naltrexone-bupropion, phentermine-topiramate, semaglutide, or tirzepatide) with lifestyle modification vs lifestyle modification alone in US adults.

## Introduction

Obesity is a complex and heterogeneous chronic disease, affecting more than two-fifths of US adults.^1,2,3,4,5^ The introduction of glucagon-like peptide-1 receptor agonists (GLP-1 RAs) for obesity treatment offers opportunities to mitigate the substantial health and economic burdens of obesity. Clinical trials of semaglutide and tirzepatide have shown substantial mean reductions in body weight (14.9% and 20.9%, respectively, compared with placebo) and cardiovascular benefits for secondary prevention.^6,7,8,9,10,11^ However, the public demand for these medications has led to supply shortages, and their high costs have led insurance companies to limit access.^12,13^

Cost-effectiveness analysis offers a standardized approach for comparing the value of new antiobesity medications. Recent cost-effectiveness analyses of the new antiobesity medications have generated conflicting findings regarding their economic value. One cost-effectiveness analysis found that semaglutide was cost-effective compared with no treatment, no lifestyle modification, or treatment with liraglutide, naltrexone-bupropion, or phentermine-topiramate.^14^ However, other cost-effectiveness analyses concluded semaglutide and tirzepatide were not cost-effective compared with lifestyle modification, primarily due to their high costs.^15,16^

The conflicting results of these prior studies are due to differences in model inputs and assumptions that require reexamination. To date, previous studies have used mean weight reduction to estimate weight-related comorbidities, failing to capture the broader cardiovascular effects of antiobesity medications and their downstream long-term potential benefits.^14,15,16^ Prior studies have also relied on imprecise or outdated antiobesity medication net price estimates, contributing to variability in incremental cost-effectiveness ratio (ICER) results.^14,15,16^ Prior cost-effectiveness analyses were also based on the characteristics of clinical trial populations, which may not reflect the real-world US population.^14,15,16^ Given all these limitations, an updated cost-effectiveness analysis to understand the health and economic implications of antiobesity medications for the US population is needed.

## Methods

We evaluated the lifetime health effects and cost-effectiveness of 4 antiobesity medications (tirzepatide, semaglutide, naltrexone-bupropion, or phentermine-topiramate) combined with lifestyle modification vs lifestyle modification alone. The University of Chicago institutional review board determined this study was not human subjects research and did not require review. The study adhered to the Consolidated Health Economic Evaluation Reporting Standards (CHEERS) reporting guideline and the Criteria for Health Economic Quality Evaluation instrument.^17,18^

### Diabetes, Obesity, Cardiovascular Disease Microsimulation Model

**Figure 1.**
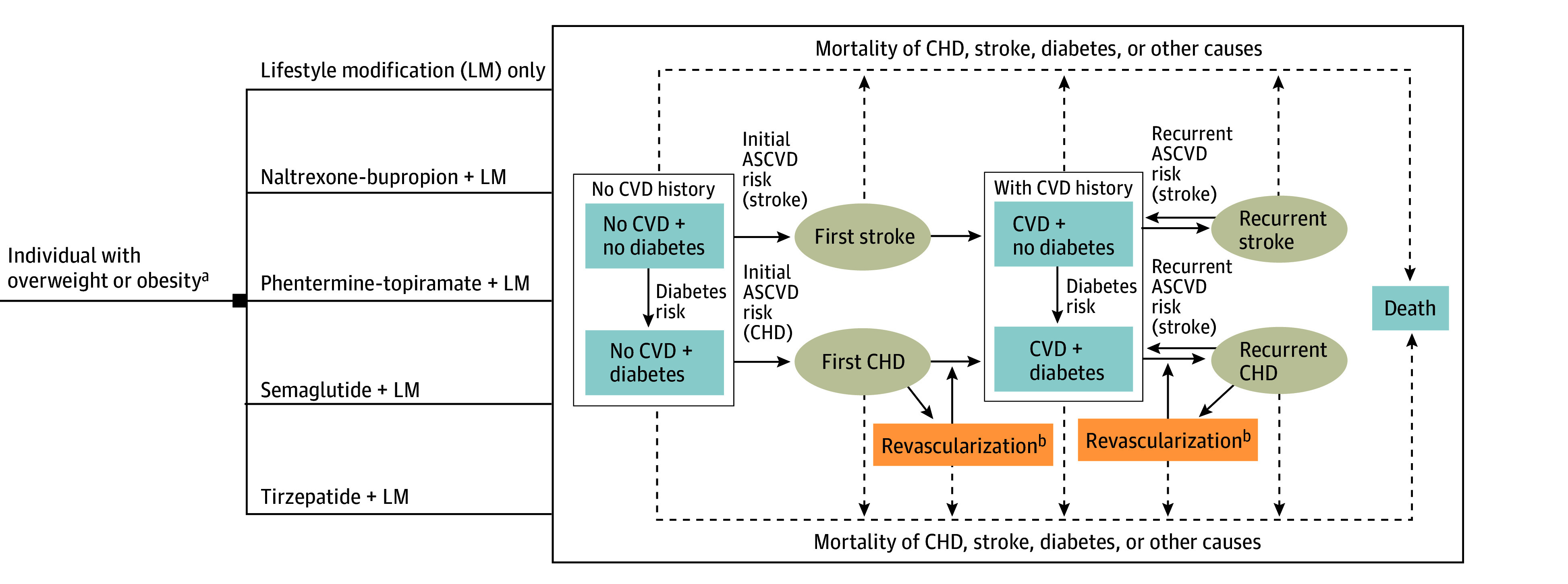
Diabetes, Obesity, Cardiovascular Disease Microsimulation Model The figure has been modified from the original framework developed by Kim et al^19^ to suit the specific context of the current study. Individuals either receive LM or an antiobesity medication and LM. The decision node (solid black square) indicates the treatment decision. The Markov model incorporates 5 health states (blue rectangles) where individuals may reside over time and link to potential cardiovascular disease (CVD) events (brown circles) occurring annually. The solid lines and arrows depict transitions between health states and events; the dotted lines and arrows indicate mortality linked to specific causes or events. The simulation continuously updates each individual’s health metrics, dictating their journey through various health states and the probability of events. ASCVD indicates atherosclerotic cardiovascular disease; CHD, coronary heart disease. ^a^Overweight was defined as a body mass index (BMI; calculated as weight in kilograms divided by height in meters squared) between 27 and 29.9 and at least 1 weight-related comorbidity. Obesity was defined as a BMI of 30 or greater. The BMI changes were separately tracked for each individual. ^b^Includes coronary artery bypass graft surgery and percutaneous coronary intervention.

We used the Diabetes, Obesity, Cardiovascular Disease Microsimulation (DOC-M) model, which is a validated probabilistic and dynamic microsimulation model that forecasts the long-term development of obesity and associated conditions, health-related quality of life, and health care costs (eMethods in Supplement 1).^19^ The model estimates annual transition probabilities among 5 health states and 4 cardiovascular disease–specific events for individuals (Figure 1) based on demographic characteristics, baseline health conditions, cardiometabolic risk factors, complication rates of health states, and population-level risk trends of these specific health states. Missing data were addressed through multiple imputations using predictive mean matching for all relevant variables (eTable 1 in Supplement 1).

We modified the DOC-M model to incorporate antiobesity medication–specific effects on weight and 6 cardiometabolic risk factors (eg, blood pressure, glucose, lipids), antiobesity medication–specific adverse events affecting quality of life, and discontinuation rates based on clinical trial evidence (eTables 2-3 in Supplement 1).^9,11,20,21,22,23,24,25^

### Population

We used data from the 2017-2020 National Health and Nutrition Examination Survey of 4823 individuals (representing 126 million eligible US adults) aged 20 to 79 years who would meet the following clinical trial inclusion criteria for these drugs: (1) had a body mass index (BMI; calculated as weight in kilograms divided by height in meters squared) of 30 or greater or (2) had a BMI between 27 and 29.9 and at least 1 weight-related comorbidity (ie, diabetes, hypertension, dyslipidemia, or cardiovascular disease).

### Treatments and Comparators

In each simulation, eligible individuals received 1 of the 4 antiobesity medications along with lifestyle modification or lifestyle modification alone.^9,11,20,21,22,23,24,25^ In the first year, we assumed that individuals experienced adverse events that led to discontinuation of the treatment at the same rates as reported in the trials. Individuals who stopped use of an antiobesity medication were assumed to continue with lifestyle modification; individuals who did not have adverse events were assumed to continue treatment indefinitely.

Lifestyle modification consisted of a hypocaloric diet with a 500 kcal/d deficit and an exercise program involving at least 150 minutes of physical activity per week, as outlined in the trial protocols.^9,24^ The antiobesity medications were (1) naltrexone-bupropion extended-release (daily oral), (2) phentermine-topiramate (daily oral), (3) semaglutide (weekly injection), or (4) tirzepatide (weekly injection).^9,11,20,21,22,23,24,25,26^

### Changes in Weight and Cardiovascular Risk Factors

We assumed that individuals receiving antiobesity medications experienced the mean changes in weight loss and 6 cardiometabolic risk factors corresponding to clinical evidence during the first 2 years.^9,11,20,21,22,23,24,26^ Beyond this period, no further changes in weight loss and cardiometabolic risk factors were expected. For individuals with diabetes, trial-specific evidence was applied.^21,22,23,24,25^ For individuals who received lifestyle modification only, they experienced changes in weight and cardiometabolic risk factors as observed in the lifestyle modification (placebo) group of the SURMOUNT tirzepatide trials.^9,24^ If individuals discontinued treatment due to adverse events, the changes in weight and cardiometabolic risk factors reverted to their levels in the second year and followed risk factor–specific temporal trends. The temporal trends were estimated from historical National Health and Nutrition Examination Survey data from 1999 to 2016 across population subgroups by age, sex, and racial and ethnic groups.^19^

### Adverse Events and Treatment Discontinuation

First-year adverse events (eg, nausea, diarrhea, vomiting, headache) were incorporated based on the trial data.^9,11,20,23^ Beyond the first year, no additional adverse events or treatment discontinuations were modeled.

### Treatment Costs

We used SSR Health data to estimate net prices for semaglutide and tirzepatide, reflecting rebates and discounts across Medicaid, the 340B drug pricing program, Medicare Part D, and commercial insurers.^27,28^ We used publicly available federal supply schedule prices for naltrexone-bupropion and phentermine-topiramate, which serve as federal benchmarks for federal purchasers.^29,30^ The net price of tirzepatide (Zepbound) was unavailable, so we estimated the relative price index of semaglutide for its diabetes (Ozempic) and obesity indications (Wegovy) and applied this index to tirzepatide (ie, Mounjaro to Zepbound).^27^ Based on the Look AHEAD study, our model assumed that individuals who discontinued antiobesity medications incurred only lifestyle modification costs.^31,32^

### Nontreatment Health Care Costs

Our model estimated annual health care costs for each individual based on age, sex, race and ethnicity, BMI, diabetes, hypertension, and cardiovascular disease using data from the 2016-2020 Medical Expenditure Panel Survey.^19^ The data from this survey include inpatient hospital stays, emergency department visits, outpatient services, prescription medications, dental care, and home health services.^33^ Cardiovascular disease–related, procedure-specific expenses were derived from mean payments in the Medicare Inpatient Prospective Payment System. All costs were adjusted to 2023 US dollars using the health care component of the Personal Consumption Expenditures price index.^34,35^

### Productivity Loss

We incorporated the economic effects of lost productivity due to morbidity and premature death associated with obesity, diabetes, and cardiovascular disease, which were derived by dividing the total national productivity loss for each condition by the projected number of US cases.^36,37,38,39^

### Quality-of-Life Adjustments

We applied individual health-related quality-of-life estimates using the US health-related quality-of-life prediction model.^19,40^ This model incorporates primary variables such as age, sex, race and ethnicity, and health conditions (eg, diabetes, hypertension, and cardiovascular disease). Event-specific, short-term reductions in health-related quality of life were applied for individuals who experienced acute coronary heart disease, stroke, or treatment-related adverse events.

### Cost-Effectiveness Analysis

We estimated the lifetime incremental health outcomes by measuring the difference in incident cases and life-years gained between individuals for lifestyle modification alone vs those using antiobesity medications with lifestyle modification. The outcomes were scaled up to 100 000 eligible individuals. We conducted the cost-effectiveness analysis in 2024 from both a US health care perspective, which included direct treatment costs and health care expenditures, and a modified societal perspective, which incorporated the productivity losses associated with disease.^41,42^ The main outcomes were discounted life-years, quality-adjusted life-years (QALYs), total costs (in 2023 US dollars), and ICERs at a 3% discount rate.^41^ We selected a threshold of less than $100 000/QALY to determine value-based price benchmarks based on prior studies.^43,44,45^

### Subgroup, Threshold, Scenario, and Sensitivity Analyses

We conducted a subgroup analysis by BMI categories, age groups, the presence of weight-related comorbidities, and diabetes status. A threshold analysis was conducted to examine the net prices at which antiobesity medications could become cost-effective. The scenario analyses included a no intervention group for comparison and 10-year treatment adherence. We also assessed the effects of changes in the annual discount and treatment discontinuation rates to reflect potentially different rates in real-world populations. To account for parameter uncertainty simultaneously, we performed probabilistic sensitivity analyses based on 1000 Monte Carlo simulations derived from probabilistic distributions for each parameter, including beta distributions for health states and specific procedures, deterministic for the pricing of antiobesity medications, normal distribution for the antiobesity medication treatment effects, uniform distribution for adverse effects, and gamma distribution for health care costs (eMethods and eTable 2 in Supplement 1).

## Results

### Baseline Characteristics

Among the 126 million eligible US adults from the National Health and Nutrition Examination Survey, the mean age was 48 years (SE, 0.5 years); 51% were female; and the initial mean BMI was 34.7 (SE, 0.2) (Table 1); and 85% had at least 1 weight-related comorbidity.

**Table 1.** Baseline Characteristics of the Cohort

	Total (N = 4823)^a^	Overweight (n = 1131)^b^	Obesity^c^		
			Class I (n = 1847)	Class II (n = 976)	Class III (n = 869)
Age, mean (SE), y	48 (0.5)	52.5 (0.8)	48.5 (0.5)	47.5 (0.7)	45.6 (0.6)
Sex, %					
Female	51.0	46.0	46.0	54.4	64.5
Male	49.0	54.0	54.0	45.6	35.5
Race and ethnicity, %^d^					
Hispanic	17.9	18.6	19.0	19.3	13.2
Non-Hispanic Black	12.8	9.8	12.0	14.5	17.1
Non-Hispanic White	60.8	59.9	61.0	59.8	63.0
Other^e^	8.5	11.7	0.8	6.4	6.7
Weight, mean (SE), kg	98.3 (0.6)	80.8 (0.5)	92.5 (0.5)	104.6 (0.6)	128.7 (1.0)
Body mass index, mean (SE)^f^	34.7 (0.2)	28.4 (0.05)	32.2 (0.05)	37.1 (0.07)	45.9 (0.3)
Blood pressure, mean (SE), mm Hg					
Systolic	123.3 (0.4)	126.5 (0.9)	122.5 (0.5)	123.9 (0.7)	120.0 (0.9)
Diastolic	76.6 (0.2)	76.3 (0.5)	76.0 (0.3)	77.8 (0.3)	77.4 (0.5)
Fasting blood glucose, mean (SE), mg/dL	114.2 (0.6)	111.9 (0.9)	112.4 (1.03)	117.1 (1.5)	117.8 (1.5)
Hemoglobin A_1c_, mean (SE), %	5.8 (0.02)	5.8 (0.03)	5.8 (0.05)	5.9 (0.04)	6.0 (0.05)
Lipid level, mean (SE), mg/dL					
High-density lipoprotein cholesterol	49.0 (0.5)	50.8 (0.9)	49.8 (0.6)	47.3 (0.6)	46.7 (0.6)
Low-density lipoprotein cholesterol	116.4 (1.1)	116.9 (1.7)	118.3 (1.4)	115.4 (2.1)	112.2 (1.8)
Total cholesterol	189.0 (1.5)	191.8 (1.8)	191.9 (1.7)	186.5 (2.1)	181.5 (2.1)
Triglycerides	119.2 (2.6)	121.8 (4.1)	119.8 (3.0)	119.6 (4.6)	113.6 (4.2)
Smoking, %	15.7	17.7	14.2	14.9	17.6
Diabetes, %^g^	17.1	15.1	14.4	20.5	22.3
Hypertension, %^h^	63.1	69.3	58.3	65.0	63.4
Hyperlipidemia, %^i^	70.5	75.0	65.4	72.7	73.6
Cardiovascular disease, %^j^	11.7	12.0	11.1	12.4	11.7
Weight-related comorbidity, %	85.0	100.0	75.3	85.6	86.4

SI conversion factors: To convert glucose to mmol/L, multiply by 0.0555; high-density and low-density lipoprotein cholesterol and total cholesterol to mmol/L, multiply by 0.0259; triglycerides to mmol/L, multiply by 0.0113.

^a^ The study cohort was selected from the 2017 to prepandemic 2020 National Health and Nutrition Examination Survey (NHANES), comprising a sample of 4823 adults. The analysis was weighted using NHANES sampling strategies to ensure it accurately reflected the demographic composition of the noninstitutionalized adult population in the US.

^b^ Defined as a body mass index between 27 and 29.9 with at least 1 weight-related comorbidity (ie, diabetes, hypertension, dyslipidemia, or cardiovascular disease).

^c^ Class I was defined as a body mass index between 30 and 34.9; class II, a body mass index between 35 and 39.9; class III, a body mass index of 40 or higher.

^d^ Collected through predefined categories.

^e^ Included non-Hispanic Asian individuals and those who selected multiple races.

^f^ Calculated as weight in kilograms divided by height in meters squared.

^g^ Self-reported diagnosis, had use of diabetes medication, had a fasting blood glucose level greater than 125 mg/dL (6.94 mmol/L), or had a hemoglobin A_1c_ value of 6.5% or greater.

^h^ Self-reported diagnosis; had use of treatment for hypertension; or had a mean systolic blood pressure of 130 mm Hg or greater or a mean diastolic blood pressure of 80 mm Hg or greater.

^i^ Self-reported diagnosis; had use of treatment for hyperlipidemia; or had a low-density protein cholesterol level greater than 160 mg/dL (8.88 mmol/L), a triglyceride level greater than 150 mg/dL (1.69 mmol/L), or a high-density lipoprotein cholesterol level less than 40 mg/dL (2.22 mmol/L) (<50 mg/dL [<2.78 mmol/L] for females).

^j^ Self-reported coronary heart disease, congestive heart failure, myocardial infarction, angina, or stroke; or had use of treatment for angina.

### Lifetime Health Outcomes

Compared with lifestyle modification alone, each of the 4 antiobesity medications with lifestyle modification would reduce rates of obesity, diabetes, and cardiovascular disease and their related deaths over the lifetime (60 years) of patients (Table 2 and eFigure 1 in Supplement 1). Compared with lifestyle modification alone, tirzepatide and lifestyle modification would reduce the highest number of obesity cases (45 609 [95% uncertainty interval {UI}, 45 092-46 126] per 100 000 eligible individuals), the number of diabetes cases (20 854 [95% UI, 19 432-22 276] per 100 000 eligible individuals), and the number of cardiovascular disease cases (10 655 [95% UI, 10 124-11 186] per 100 000 eligible individuals). In contrast, naltrexone-bupropion and lifestyle modification (compared with lifestyle modification alone) would have the smallest decrease in the number of obesity cases (6514 [95% UI, 5741-7287] per 100 000 eligible individuals), the number of diabetes cases (11 472 [95% UI, 10 594-12 350] per 100 000 eligible individuals), and the number of cardiovascular disease cases (2468 [95% UI, 2401-2534] per 100 000 eligible individuals). Compared with lifestyle modification alone, tirzepatide and lifestyle modification would also have the largest effects on reducing secondary outcomes (including first and second incident cases of cardiovascular disease and diabetes-related and cardiovascular disease–related deaths) followed by semaglutide, phentermine-topiramate, and naltrexone-bupropion, all with lifestyle modification.

**Table 2.** Estimated Health Outcomes and Cost-Effectiveness of Antiobesity Medications Over Lifetime for National Health and Nutrition Examination Survey Participants

	Mean (95% UI)^a^				
	Lifestyle modification alone	Lifestyle modification and medication			
		Naltrexone-bupropion	Phentermine-topiramate	Semaglutide	Tirzepatide
**Health outcome measures** ^b^					
Obesity^c^	[Reference]	6514 (5741 to 7287)	17 098 (15 240 to 18 956)	32 087 (31 292 to 32 882)	45 609 (45 092 to 46 126)
Diabetes	[Reference]	11 472 (10 594 to 12 350)	8266 (7637 to 8894)	19 211 (17 878 to 20 544)	20 854 (19 432 to 22 276)
Cardiovascular disease	[Reference]	2468 (2401 to 2534)	4580 (4440 to 4721)	8263 (7738 to 8788)	10 655 (10 124 to 11 186)
First incident	[Reference]	984 (965 to 1003)	2987 (2967 to 3008)	5385 (5308 to 5461)	7323 (7244 to 7402)
Second incident	[Reference]	2410 (2320 to 2500)	4966 (4789 to 5144)	7101 (6832 to 7370)	10 067 (9640 to 10 494)
Death					
Diabetes	[Reference]	654 (605 to 703)	449 (432 to 466)	1271 (1182 to 1361)	1495 (1401 to 1589)
Cardiovascular disease	[Reference]	279 (276 to 282)	1186 (1184 to 1189)	2055 (2029 to 2081)	2897 (2882 to 2911)
Life-years gained	[Reference]	11 406 (10 179 to 12 632)	20 153 (18 287 to 22 018)	35 634 (34 006 to 37 262)	48 649 (45 744 to 51 554)
**Cost-effectiveness measures**					
Life-years	30.47 (29.72 to 31.22)	30.58 (29.82 to 31.35)	30.67 (29.90 to 31.43)	30.82 (30.05 to 31.60)	30.97 (30.20 to 31.74)
Incremental life-years	[Reference]	0.11	0.20	0.35	0.50
QALY	16.38 (16.06 to 16.70)	16.44 (16.12 to 16.76)	16.52 (16.20 to 16.85)	16.63 (16.30 to 16.96)	16.73 (16.41 to 17.05)
Incremental QALY	[Reference]	0.06	0.14	0.25	0.35
Costs, $					
Lifestyle modification	31 052 (30 517 to 31 587)	31 894 (31 361 to 32 426)	31 954 (31 419 to 32 490)	32 039 (31 496 to 32 582)	32 134 (31 599 to 32 670)
Medication	NA	6498 (6379 to 6616)	27 751 (27 249 to 28 254)	148 897 (146 318 to 151 476)	111 610 (109 738 to 113 482)
Health care expenditure	182 425 (177 303 to 187 547)	175 947 (170 838 to 181 055)	171 637 (166 511 to 176 764)	160 974 (156 125 to 165 823)	154 028 (149 226 to 158 829)
Loss of productivity	30 854 (28 570 to 33 137)	27 211 (24 936 to 29 486)	24 921 (22 601 to 27 241)	19 340 (17 443 to 21 238)	15 517 (13 774 to 17 260)
Total	244 331	241 550	256 263	361 250	313 289
Incremental cost	[Reference]	−2781	11 932	116 919	68 958
Incremental cost-effectiveness ratio, $ per QALY gained^d^	[Reference]	Cost saving	85 229	467 676	197 023

Abbreviations: NA, not applicable; QALY, quality-adjusted life-year; UI, uncertainty interval.

^a^ Unless otherwise indicated.

^b^ Data are expressed as estimated cases averted per eligible 100 000 population (calculated the difference in cumulative incidence of health outcomes between the lifestyle modification and antiobesity medication interventions). This difference was multiplied by the total population size and then scaled per 100 000 eligible population.

^c^ A body mass index (calculated as weight in kilograms divided by height in meters squared) of 30 or greater.

^d^ Calculated as the estimated mean net change in costs from a health care perspective divided by the mean net change in QALYs. Amounts below $100 000/QALY gained were considered cost-effective.

The 4 antiobesity medications, all with lifestyle modification, increased life expectancy and QALY gained to varying degrees compared with lifestyle modification alone (Table 2). Compared with lifestyle modification alone, tirzepatide and lifestyle modification generated 48 649 (95% UI, 45 744-51 554) life-years gained per 100 000 eligible population and semaglutide and lifestyle modification generated 35 634 (95% UI, 34 006-37 262) life-years gained per 100 000 eligible population. Compared with lifestyle modification alone, phentermine-topiramate and lifestyle modification generated 20 153 (95% UI, 18 287-22 018) life-years gained per 100 000 eligible population and naltrexone-bupropion and lifestyle modification generated 11 406 (95% UI, 10 179-12 632) life-years gained per 100 000 eligible population.

### Lifetime Costs and Cost-Effectiveness

Compared with lifestyle modification alone, tirzepatide and lifestyle modification and semaglutide and lifestyle modification provided long-term health care cost savings and the lowest levels of productivity loss due to improved health outcomes, but the high costs of tirzepatide and semaglutide offset these savings. Compared with lifestyle modification alone, tirzepatide and lifestyle modification had the lowest mean per-person background health care expenditures at $154 028, followed by semaglutide and lifestyle modification at $160 974 (Table 2). However, due to their high treatment costs, the ICER was $197 023/QALY gained for tirzepatide and lifestyle modification and was $467 676/QALY gained for semaglutide and lifestyle modification. Compared with lifestyle modification alone, naltrexone-bupropion and lifestyle modification was cost saving because of its lower treatment costs. In addition to being more effective than naltrexone-bupropion and lifestyle modification, phentermine-topiramate and lifestyle modification had an ICER of $85 229/QALY gained.

### Subgroup Analyses

The BMI subgroup analysis (overweight and obesity categories) revealed no consistent pattern in health and economic outcomes across categories (eg, the number of cases averted does not systematically increase or decrease across the categories from lowest to highest) (eTables 4-5 in Supplement 1). Among all antiobesity medications, the combination of naltrexone-bupropion with lifestyle modification was consistently cost saving across all overweight and obesity categories compared with lifestyle modification only. For obesity class I, phentermine-topiramate and lifestyle modification had an ICER of $33 005/QALY gained, indicating cost-effectiveness (eTable 5 in Supplement 1).

The subgroup analysis based on the presence of comorbidities revealed individuals with comorbidities had greater incremental gains in QALY, indicating that these interventions may offer more benefits for those with existing weight-related conditions (eTable 6 in Supplement 1). Naltrexone-bupropion and lifestyle modification was cost saving in the group with comorbidities and in the group without comorbidities, whereas phentermine-topiramate and lifestyle modification had the lowest ICER of $110 600/QALY gained in the group with comorbidities.

The subgroup analysis based on diabetes status showed that the lower cost-effectiveness for individuals with diabetes (vs those without diabetes) can be attributed to the relatively lower percentage of weight loss and less improvement in 6 cardiometabolic risk factors from treatment with antiobesity medications (eTable 7 in Supplement 1). Additional analyses based on age groups appear in eTable 8 in Supplement 1.

### Threshold Analysis

The threshold analysis suggested that tirzepatide and lifestyle modification would need a 30.5% additional reduction from its estimated current net price^27,28,29,30^ of $6236 to an annual price of $4334 to become cost-effective. Semaglutide and lifestyle modification would become cost-effective with an 81.9% discount from its estimated current net price of $8412 to an annual price of $1522 (Table 3 and eFigure 2 in Supplement 1). The list prices^46,47,48,49^ also are listed in Table 3.

**Table 3.** Threshold Analysis of Antiobesity Medication Costs

	Naltrexone-bupropion	Phentermine-topiramate	Semaglutide	Tirzepatide
**List price**				
Annual price, $^46,47,48,49^	3598	2786	16 188	12 718
Discount, %^a^				
$100 000/QALY	72.4	30.8	90.6	65.9
$150 000/QALY	67.0	14.6	86.1	58.2
$200 000/QALY	61.6	NA	81.8	50.5
Annual cost, $^b^				
$100 000/QALY	993	1928	1522	4334
$150 000/QALY	1197	2379	2246	5313
$200 000/QALY	1381	NA	2953	6295
**Net price**				
Annual price, $^27,30^	421	1786	8412	6236
Discount, %^a^				
$100 000/QALY	NA	NA	81.9	30.5
$150 000/QALY	NA	NA	73.3	14.8
$200 000/QALY	NA	NA	64.9	NA
Annual cost, $^b^				
$100 000/QALY	NA	NA	1522	4334
$150 000/QALY	NA	NA	2246	5313
$200 000/QALY	NA	NA	2953	NA

Abbreviations: NA, not applicable; QALY, quality-adjusted life-year.

^a^ This is the percentage discount required on the list price or net price to achieve the predefined QALY target.

^b^ This is the price after discounts and rebates were applied and was used to assess the cost-effectiveness of each antiobesity medication.

### Scenario Analyses

In the scenario analysis comparing antiobesity medications and lifestyle modification vs no intervention, even though all 4 antiobesity medications improved health outcomes when combined with lifestyle modification, none were found to be cost-effective over a lifetime (eTable 9 in Supplement 1).

As the discontinuation rate increased from 5% to 50%, the treatment costs for the antiobesity medications were reduced, but the incremental gains in QALYs declined, leading to less favorable ICERs across all antiobesity medications (eTable 10 in Supplement 1). Additional analyses based on 10-year treatment adherence appear in eTable 11 in Supplement 1.

### Sensitivity Analyses

**Figure 2.**
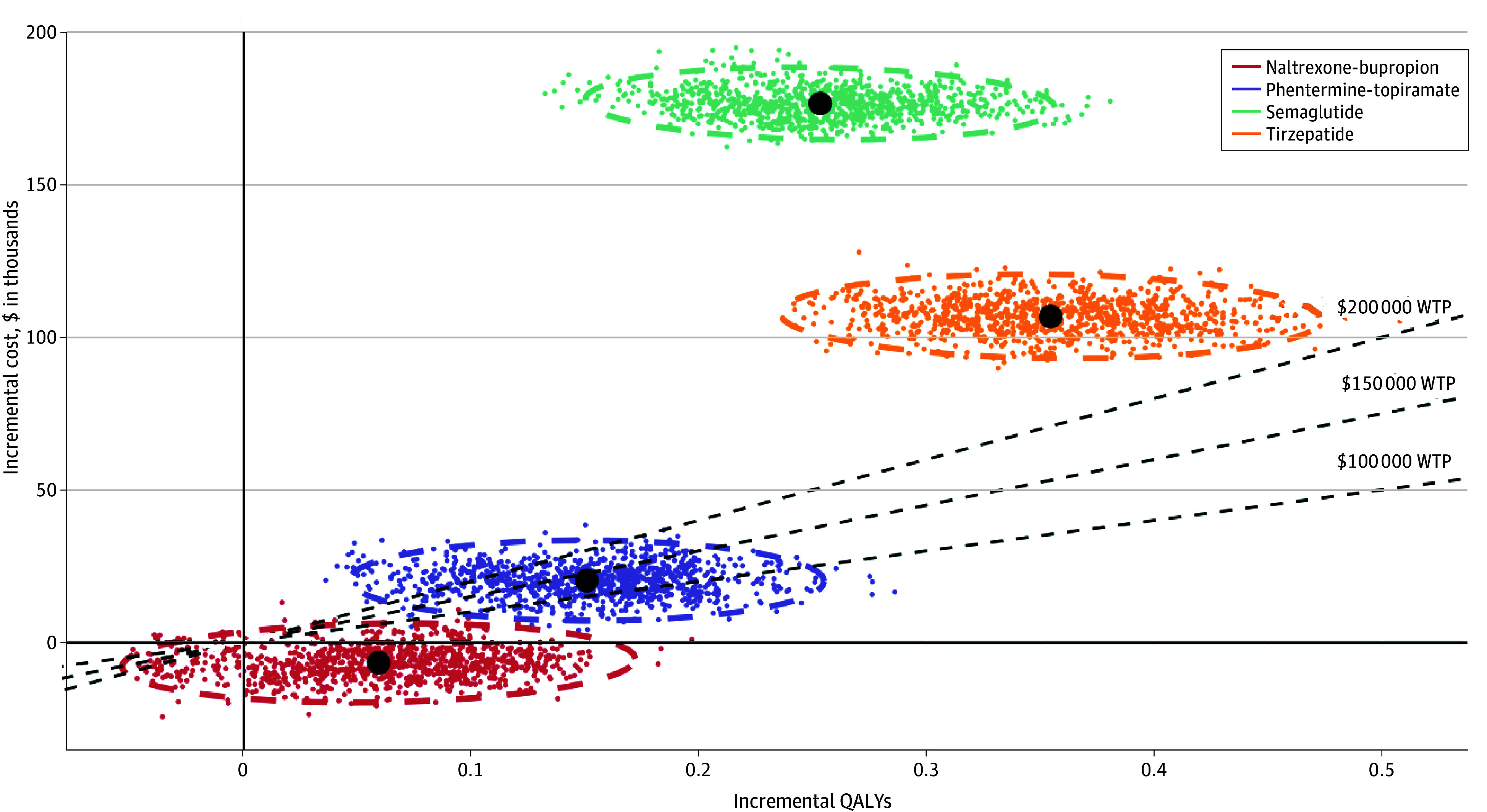
Probabilistic Sensitivity Analysis for the Cost-Effectiveness of the Antiobesity Medications vs Lifestyle Modification Over a Lifetime Each data point represents 1 of 1000 Monte Carlo simulations, and the encompassing ellipses illustrate the 95% uncertainty intervals for these results. The solid black circles indicate the mean values for the 1000 simulations. The willingness-to-pay (WTP) thresholds of $100 000, $150 000, and $200 000 per quality-adjusted life-year (QALY) are depicted by dashed lines.

The probability of naltrexone-bupropion and lifestyle modification being cost-effective was 89.1% at $100 000/QALY, increasing to 91.2% at $200 000/QALY. Phentermine-topiramate and lifestyle modification had a 23.5% probability of cost-effectiveness at $100 000/QALY, increasing to 83.4% at $200 000/QALY. Semaglutide and lifestyle modification and tirzepatide and lifestyle modification had a 0% probability of cost-effectiveness across all thresholds (Figure 2 and eTable 12 in Supplement 1).

Across all antiobesity medications, a higher discount rate at 5% led to less favorable cost-effectiveness ratios because of greater reductions in discounted QALYs gained than those in discounted costs (eTable 13 in Supplement 1). Naltrexone-bupropion and lifestyle modification was health improving and cost saving compared with lifestyle modification only at any discount rate.

## Discussion

The current study provides evidence on the long-term health effects and cost-effectiveness of 4 antiobesity medications, accounting for the interplay between obesity, diabetes, and cardiovascular disease while incorporating updated data on the cardiovascular benefits of antiobesity medications and net prices in a representative US population. Tirzepatide and semaglutide, both with lifestyle modification, generate the greatest improvements in quality-adjusted life expectancy. However, both antiobesity medications and lifestyle modification were not cost-effective at the $100 000/QALY benchmark due to their high lifetime treatment costs. The threshold analysis indicates that a substantial price reduction (30.5% for tirzepatide and 81.9% for semaglutide) would be needed to meet a cost-effectiveness threshold of $100 000/QALY.

Prior cost-effectiveness analyses of the new antiobesity medications have generally concluded that agents (such as tirzepatide and semaglutide) combined with lifestyle modification were not cost-effective, but there are significant differences in the ICER estimates due to variation in the model inputs and assumptions.^14,15,16^ For semaglutide and lifestyle modification, Kim et al^14^ reported an ICER of $123 000/QALY compared with lifestyle modification alone, whereas the Institute for Clinical and Economic Review^16^ reported an ICER of $237 000/QALY compared with lifestyle modification alone. Kim et al^14^ reported a lower ICER because the study only considered 2 years of treatment and not lifelong treatment with semaglutide. The study by Gómez Lumbreras et al^15^ reported an ICER of $24 million/QALY for semaglutide and lifestyle modification vs phentermine-topiramate and lifestyle modification. The estimate from the current study for the same comparison was much lower at $100 000/QALY than the estimate from Gómez Lumbreras et al,^15^ likely due to the incorporation of broader long-term benefits with semaglutide. For tirzepatide and lifestyle modification, Gómez Lumbreras et al^15^ found that the drug had the greatest increase in QALYs among antiobesity medications with an ICER of $356 000/QALY compared with phentermine-topiramate and lifestyle modification. The current study also found that tirzepatide and lifestyle modification generated a greater improvement in QALYs, and the ICER estimate ($197 023/QALY) was lower.

A critical difference across studies is the assumed price of antiobesity medications. We used the net price, a byproduct of negotiations between the drug manufacturers, pharmacy benefit plans, and insurers that affect how drug prices are discounted. Net prices are based on these discounts that are inconsistent across payers, and net prices are highest for those who are uninsured or insured without coverage.^27,50^ Most state Medicaid programs and Medicare do not cover antiobesity medications, and private insurance plans restrict access by requiring prior authorization.^51,52^ Consequently, the lack of additional discounts and inadequate coverage through state, federal, and private insurance programs leave many patients unable to afford these medications, resulting in higher premiums and cost sharing.^53^ These high net prices will likely exacerbate obesity disparities, disproportionately affecting racial and ethnic groups with unequal health care access.^54,55,56^

Policy solutions are crucial to improve antiobesity medication affordability and access. Since the US Food and Drug Administration approved the first GLP-1 RA nearly 2 decades ago, only 1 generic GLP-1 RA has been approved,^57^ primarily due to extensive patent and regulatory strategies that significantly delay generic entry.^58^ Although the Hatch-Waxman Act was implemented to increase generic drug availability, increase competition, and reduce costs, this act is less effective for drug delivery devices necessary for many of the GLP-1 RAs.^58,59^ Increased market competition is anticipated, but further policy reforms to the patent system and regulatory frameworks could facilitate faster entry of generic alternatives, driving down costs.^60^

In addition, policy efforts to lower prices through negotiations between payers and pharmaceutical manufacturers have taken on new life with the Inflation Reduction Act (IRA). Under the IRA, the Centers for Medicare & Medicaid Services has initiated price negotiations for selected drugs with an effective date of January 2026. Although the newer antiobesity medications were not part of the initial 10 drugs for the inaugural drug price negotiation program, semaglutide is likely to be selected for Medicare price negotiation as early as 2027 under the IRA, given its significant clinical and fiscal effects.^61,62,63^ Although the exact discount from Medicare price negotiation is uncertain, this will likely affect future prices of antiobesity medications. Moreover, a recent Centers for Medicare & Medicaid Services proposal to include antiobesity medications under Medicare Part D and Medicaid could expand access to millions of individuals.^64^ However, the Congressional Budget Office estimates that such coverage would increase federal spending by $35 billion between 2026 and 2034, raising fiscal concerns.^65^

Lastly, exploring alternative weight maintenance approaches (such as lifestyle modification programs, food is medicine interventions, and lower-dose maintenance GLP-1 RAs) after the initial use of GLP-1 RAs to achieve maximum weight loss could offer cost-effective alternatives while helping patients sustain meaningful clinical benefits.^66^ Although these alternatives potentially yield fewer health gains than antiobesity medications alone, they may result in substantial long-term savings in health care expenditures. Therefore, consideration of such programs, alongside ongoing drug pricing reforms, is essential in improving the affordability and accessibility of obesity treatment. Additionally, although compounded GLP-1 RAs are available at lower prices, their safety and efficacy remain unverified. Future research should rigorously evaluate the safety, efficacy, and cost-effectiveness of these alternatives compared with treatments approved by the US Food and Drug Administration.

### Limitations

Our study has limitations. First, uncertainty remains regarding long-term real-world clinical effectiveness, adverse event rates, adherence, and net prices of antiobesity medications. Our model may overestimate the benefits by projecting short-term clinical trial effects over a lifetime and assuming concurrent lifestyle modification. Importantly, the trial data on antiobesity medication effectiveness combined both medication and lifestyle modification, making it difficult to isolate the effects of the medications alone.

Second, differences in pricing structures introduce variability in cost estimates. The SSR Health net prices for GLP-1 RAs may underrepresent costs by excluding some supplemental rebates, whereas federal supply schedule prices for non–GLP-1 RAs may overestimate costs by excluding other broader discounts (eg, additional discounts for US Department of Veterans Affairs national contracts). Future studies using real-world data on weight trends, cardiometabolic risk, adherence patterns, and updated pricing will provide more accurate estimates of antiobesity medication cost-effectiveness and health benefits.

Third, the current DOC-M model does not explicitly incorporate diabetes-related microvascular complications (such as retinopathy, neuropathy, and nephropathy), but captures these effects indirectly through diabetes-related disutility and costs. Although this could underestimate the benefits, this approach was consistently applied across comparisons, minimizing effects on ICERs. The DOC-M model also excluded other weight-related comorbidities, such as osteoarthritis and obstructive sleep apnea. Accounting for the long-term health benefits from reducing these comorbidities with antiobesity medications might improve their cost-effectiveness.

## Conclusions

This economic evaluation found that although tirzepatide and semaglutide offered substantial long-term health benefits, they were not cost-effective at current net prices. Efforts to reduce the net prices of new antiobesity medications are essential to ensure equitable access to highly effective antiobesity medications.

## Notes

Supplement 1.
eMethods
**eTable 1.** Percentages of Missing Values for Individual-Level Characteristics in the Model Population **eTable 2.** Key Model Inputs **eTable 3.** Treatment Efficacy of Lifestyle Modifications and Anti-Obesity Medications **eTable 4.** Estimated Lifetime Health Outcomes of Anti-Obesity Medications for NHANES Participants, by BMI Categories **eTable 5.** Cost-Effectiveness Results of Lifestyle Modifications and Anti-Obesity Medications for NHANES Participants Over a Lifetime, by BMI Categories **eTable 6.** Cost-Effectiveness Results of Lifestyle Modifications and Anti-Obesity Medications Based on Comorbidities **eTable 7.** Cost-Effectiveness Results of Lifestyle Modifications and Anti-Obesity Medications Based on Diabetes Status **eTable 8.** Cost-Effectiveness Results of Lifestyle Modifications and Anti-Obesity Medications Based on Age Groups **eTable 9.** Cost-Effectiveness Results of No intervention Vs. Anti-Obesity Medications **eTable 10.** Cost-Effectiveness Results of Lifestyle Modifications and Anti-Obesity Medications Varying Treatment Discontinuation Rate **eTable 11.** Cost-Effectiveness Results of Lifestyle Modifications and Anti-Obesity Medications with 10-year of Treatment Adherence **eTable 12.** Percentage of Simulations Achieving Cost-Effectiveness at Various Willingness-to-Pay Thresholds **eTable 13.** Cost-Effectiveness Results of Lifestyle Modifications and Anti-Obesity Medications Varying Annual Discount Rate **eFigure 1.** Estimated Lifetime Health Outcomes of Anti-Obesity Medications for NHANES Participants **eFigure 2.** Threshold Analysis for Annual Net Cost of Tirzepatide and Semaglutide
eReferences

Supplement 2.
Data sharing statement
