# Supplementary material for: Lifetime Health Effects and Cost-Effectiveness of Tirzepatide and Semaglutide in US Adults
Source: JAMA Health Forum. 2025 Mar 14;6(3):e245586. doi: 10.1001/jamahealthforum.2024.5586 (PMC11909610; doi:10.1001/jamahealthforum.2024.5586)
Supplement: Supplement 1. — eMethods eTable 1. Percentages of Missing Values for Individual-Level Characteristics in the Model Population eTable 2. Key Model Inputs eTable 3. Treatment Efficacy of Lifestyle Modifications and Anti-Obesity Medications eTable 4. Estimated Lifetime Health Outcomes of Anti-Obesity Medications for NHANES Participants, by BMI Categories eTable 5. Cost-Effectiveness Results of Lifestyle Modifications and Anti-Obesity Medications for NHANES Participants Over a Lifetime, by BMI Categories eTable 6. Cost-Effectiveness Results of Lifestyle Modifications and Anti-Obesity Medications Based on Comorbidities eTable 7. Cost-Effectiveness Results of Lifestyle Modifications and Anti-Obesity Medications Based on Diabetes Status eTable 8. Cost-Effectiveness Results of Lifestyle Modifications and Anti-Obesity Medications Based on Age Groups eTable 9. Cost-Effectiveness Results of No intervention Vs. Anti-Obesity Medications eTable 10. Cost-Effectiveness Results of Lifestyle Modifications and Anti-Obesity Medications Varying Treatment Discontinuation Rate eTable 11. Cost-Effectiveness Results of Lifestyle Modifications and Anti-Obesity Medications with 10-year of Treatment Adherence eTable 12. Percentage of Simulations Achieving Cost-Effectiveness at Various Willingness-to-Pay Thresholds eTable 13. Cost-Effectiveness Results of Lifestyle Modifications and Anti-Obesity Medications Varying Annual Discount Rate eFigure 1. Estimated Lifetime Health Outcomes of Anti-Obesity Medications for NHANES Participants eFigure 2. Threshold Analysis for Annual Net Cost of Tirzepatide and Semaglutide eReferences [file jamahealthforum-e245586-s001.pdf]

Supplemental Online Content

Hwang JH, Laiteerapong N, Huang ES, Kim DD. Lifetime health effects and cost-effectiveness of tirzepatide and semaglutide in US adults. Published online March 14, 2025. *JAMA Health Forum*.
doi:10.1001/jamahealthforum.2024.5586

eMethods
eTable 1. Percentages of Missing Values for Individual-Level Characteristics in the Model Population
eTable 2. Key Model Inputs
eTable 3. Treatment Efficacy of Lifestyle Modifications and Anti-Obesity Medications
eTable 4. Estimated Lifetime Health Outcomes of Anti-Obesity Medications for NHANES Participants, by BMI Categories
eTable 5. Cost-Effectiveness Results of Lifestyle Modifications and Anti-Obesity Medications for NHANES Participants Over a Lifetime, by BMI Categories
eTable 6. Cost-Effectiveness Results of Lifestyle Modifications and Anti-Obesity Medications Based on Comorbidities
eTable 7. Cost-Effectiveness Results of Lifestyle Modifications and Anti-Obesity Medications Based on Diabetes Status
eTable 8. Cost-Effectiveness Results of Lifestyle Modifications and Anti-Obesity Medications Based on Age Groups
eTable 9. Cost-Effectiveness Results of No intervention Vs. Anti-Obesity Medications
eTable 10. Cost-Effectiveness Results of Lifestyle Modifications and Anti-Obesity Medications Varying Treatment Discontinuation Rate
eTable 11. Cost-Effectiveness Results of Lifestyle Modifications and Anti-Obesity Medications with 10-year of Treatment Adherence
eTable 12. Percentage of Simulations Achieving Cost-Effectiveness at Various Willingness-to-Pay Thresholds
eTable 13. Cost-Effectiveness Results of Lifestyle Modifications and Anti-Obesity Medications Varying Annual Discount Rate
eFigure 1. Estimated Lifetime Health Outcomes of Anti-Obesity Medications for NHANES Participants
eFigure 2. Threshold Analysis for Annual Net Cost of Tirzepatide and Semaglutide
eReferences

This supplemental material has been provided by the authors to give readers additional information about their work.

© 2025 Hwang JH et al. *JAMA Health Forum.*

eMethods

1.1 The Diabetes, Obesity, Cardiovascular Disease Microsimulation (DOC-M) model

The Diabetes, Obesity, Cardiovascular Disease Microsimulation (DOC-M) model is a validated individual-level, health-state transition model programmed in R Statistical Software (version 4.1.0; R Core Team 2021).[1]

The model utilized data from National Health and Nutrition Examination Survey (NHANES) from 1999 to 2016. This data included demographic, cardiometabolic risk factors and related health conditions to provide estimates to predict annual risks of obesity, diabetes, first atherosclerotic cardiovascular disease (ASCVD) and second ASCVD.

The annual incidence of diabetes was estimated using the Framingham Offspring Study 8-year risk prediction model based systolic blood pressure (SBP), fasting blood glucose, body-mass index (BMI), high-density lipoprotein (HDL-C), triglycerides, history of hypertension treatment, and parental history of diabetes.[2] The first ASCVD event was estimated using the American College of Cardiology/American Heart Association (ACC/AHA) 10-year ASCVD risk equation.[3] For second ASCVD, the Framingham Heart Study coronary risk model was used to estimate 2-year risk based on age, SBP, total cholesterol, smoking status, and diabetes status.[4]

For mortality estimates, the model extracted cause-specific mortality data for the years 2012-2016 from CDC Wonder.[5] This data included information on the underlying cause of death and demographic data, stratified by age groups, sex, and four race and ethnicity groups. The model assumed that cause-specific mortalities (e.g., those from ischemic heart disease, stroke, and diabetes) were only applicable to individuals with the respective conditions (cardiovascular disease [CVD] history, first or second CVD, and diabetes), rather than the general population.

The model also effectively tracks rates of obesity (BMI $\geq$30 kg/m^2). It achieves by dynamically tracking the variations in BMI for each individual, taking into account their age, sex, and racial and ethnic to provide U.S. representative estimates. Based health outcomes, the model estimates quality-adjusted life years (QALYs) and health care costs, providing a detailed individual-level analysis.

Overall, the model enables to evaluate various health policies and interventions by projecting obesity, diabetes, and CVD-related health outcomes, along with their associated complications, health-related quality of life and health care costs. The source code for the original DOC-M is available at https://github.com/food-price/DOC-M-Model-Development-and-Validation.

1.2 Time Horizon

We assessed the cost-effectiveness of lifestyle modification alone and adjunct to anti-obesity medications (AOMs) over the lifetime (60 years).

The major clinical trials for each AOM varied in duration; however, they all extended for slightly more than one year. The naltrexone and bupropion trial, conducted by Greenway et al, spanned 56 weeks, while the phentermine and topiramate study extended over 108 weeks.[6,7] The semaglutide trial led by Wilding et al was conducted for a duration of 68 weeks, and the tirzepatide trial by Jastreboff et al lasted for 72 weeks.[8,9] Notably, to date, there are no available long-term data on AOMs extending beyond 5 years.[10]

© 2025 Hwang JH et al. *JAMA Health Forum.*

While there is limited long-term follow-up data on AOMs to evaluate whether treatment leads to sustained weight reduction, we believe that a lifetime horizon is appropriate for our analysis for the following reasons: 1) obesity is a chronic disease that requires long-term treatment and management, and 2) by simulating under the maximum treatment duration, we can project the related long-term health outcomes.

1.3 Projection of Weight and Cardiometabolic Risk Factor Changes

We utilized data from current clinical trials to extrapolate changes in weight and related cardiometabolic risk factors, including SBP and diastolic blood pressure (DBP), fasting blood glucose, total cholesterol, HDL-C, and triglycerides, aiming to understand how changes in these cardiometabolic risk factors due to AOMs impact long-term health outcomes.[6-9,11-13] The treatment-induced weight reduction and changes in cardiometabolic risk factors were calibrated to reflect the intention-to-treat analysis of relative changes from baseline, considering the proportion of individuals who discontinued treatment.

To model the impact of lifestyle modifications on weight and cardiometabolic risk factors, we referenced the placebo group outcomes from the SURMOUNT-1 trial, led by Jastreboff et al, ensuring consistency in our approach.[8] When diabetes-specific data from clinical trials was available, it was utilized in preference for individuals with diabetes.[7,11-14]

Given the lack of long-term data on weight and cardiometabolic risk factor changes and considering that trials exceeding 52 weeks did not demonstrate substantial changes, we have presumed that weight and cardiometabolic risk factors would not further improve after the first year.[15-19] Therefore, we maintained the initial changes achieved with AOMs throughout the treatment period in our projections.

1.4 Change in Weight and Cardiometabolic Risk Factors after Treatment Discontinuation

Projection of weight regain after an individual discontinued treatment was modeled based on the clinical trials by Wilding et al and Aronne et al.[9,16,19] In a STEP-1 extension analysis led by Wilding et al, semaglutide led to a mean weight loss of 17.3% over 68 weeks, which decreased to a net loss of 5.6% at week 120 after treatment cessation, reflecting a significant weight regain and a reversion of cardiometabolic gains.[16] Similarly, an 88-week study by Aronne et al found that participants who discontinued tirzepatide treatment after an initial 36 weeks experienced 14% weight regain after the treatment discontinuation.[19] Based on these findings, individuals were assumed to revert to their baseline weight and cardiometabolic risk factors by the end of the second year following cessation of treatment. Individuals adhering to lifestyle modifications only followed natural trends in weight and cardiometabolic risk factors after the discontinuation.

Those treated with both AOMs and lifestyle modifications, who later discontinued the AOM, continued to follow the weight and cardiometabolic risk factor trends associated with lifestyle modification alone.

1.5 Adverse Events and Probability of Treatment Discontinuation

We estimated common adverse events (AEs) and AEs attributable to AOM treatment discontinuation based on the clinical trials.[6-9,11,12] When specific AE rates were available for different doses, we incorporated highest dose-specific AE rates into our model. From the cohort of individuals receiving AOM treatment in our simulation, we randomly selected a subset who experienced AEs. Within this subset, we further identified individuals who discontinued treatment, according to the reported AE-related discontinuation rates for each AOM. Given that reported AEs primarily occurred during the initial dose titration period, we assumed that individuals who did not experience AEs during the first year would not encounter significant AEs, and therefore, no further AEs were modeled beyond this period.[6-8]

1.6 Stakeholder Engagement

The research team members have clinical expertise in managing obesity, diabetes, and CVD. This practical experience provided valuable insights into the treatment outcomes and challenges faced by patients taking AOMs. The team's direct patient care experience ensured that the study design, assumptions, modeling parameters, and cost estimates were highly relevant to real-world clinical settings. The study design also drew on established clinical guidelines and literature, which reflect broad expert consensus and patient care standards in obesity treatment. Regular internal reviews and discussions among the research team, based on their collective clinical experience, guided the refinement of the study protocols and assumptions.

1.7 Health-Related Quality of Life

The DOC-M estimated individual health-related quality of life estimates using the US health-related quality of life (HRQOL) prediction model, developed from a self-administered EuroQol 5 Dimension 5 Level (EQ-5D) questionnaire completed by 13646 adults in the 2000 Medical Expenditure Panel Survey—a nationally representative US sample.[20,21] This model incorporated primary variables such as age, sex, race and ethnicity, and health conditions, (e.g., diabetes, hypertension, and CVD) without assigning separate utility values for BMI categories. Event-specific short-term reduction in HRQOL were applied for individuals who experienced acute coronary heart disease or stroke. For each type of adverse event associated with AOMs, we applied event-specific disutility values. To reflect the uncertainty of these measures, we introduced variability by simulating disutility using a beta distribution, defined to 20% of the respective disutility estimates.

We referenced the disutility values from individuals with type 2 diabetes, migraines, and chronic headaches due to limited data on the disutility resulting from AEs associated with AOMs. [22-24] For common AEs like paresthesia, dry mouth, and constipation associated with phentermine and topiramate, we applied disutility measures linked with preventive migraine treatments.[25] The disutility from adverse gastrointestinal effects, such as nausea, vomiting, and diarrhea, were estimated from various health states in individuals with type 2 diabetes.[26]

1.8 Estimated Costs

© 2025 Hwang JH et al. *JAMA Health Forum.*

We estimated cost of treatment from various sources. To maintain consistency with the clinical trial data, we derived the intensive lifestyle intervention costs from the trial data. Since we are projecting costs over a lifetime (60 years), we prioritized using the data with the longest time available. The Look AHEAD (Action for Health in Diabetes) cost-effectiveness study provided nine years of data, so we calculated the average annualized cost and applied it to our model. The lifestyle modification cost was derived from the intensive lifestyle intervention in the Look AHEAD study, which initially reported a cost of $11275 (2012 dollars) over nine years, or $1252 per year. This value was inflated to $1678 per year in 2023 dollars for our model.[27,28] We recognized the Look AHEAD trial is an impactful model, which integrates diet, physical activity, and behavioral changes to promote healthier lifestyles and would provide the best estimate for our study.

In our base case analysis, we calculated the net prices for AOMs from the U.S. Department of Veterans Affairs Federal Supply Schedule Service (FSS) database, and by data from SSR Health. Given the absence of recent FSS data for newer drugs like tirzepatide and semaglutide, we relied on SSR Health data to capture their net prices. SSR Health provides important data reflecting net prices across private insurers and public programs, inclusive of rebates and discounts. The absence of FSS prices for these newer medications makes SSR Health data a reasonable and valid estimate for current net prices.

For older medications such as naltrexone/bupropion and phentermine/topiramate, which are available in the FSS database, we utilized their more stable and consistent FSS pricing.[29,30] Due to their longer market presence, these prices experience fewer fluctuations, making them appropriate for representing government-negotiated price points commonly used by public agencies, such as the United States Department of Veterans Affairs (VA). This stability ensures that FSS prices serve as a strong benchmark, especially in comparison to the newer AOMs.

For tirzepatide's net price estimation, we adopted a cost extrapolation strategy, utilizing semaglutide’s pricing differentials between diabetes and obesity indications as a model for tirzepatide. Given the complex nature of the U.S. payment and reimbursement systems, which introduces significant price variability and uncertainty, these analyses aimed to align with the most up-to-date net prices.[30,31] We did not include obesity monitoring cost addition to AOM cost.

We estimated annual healthcare costs for individuals based on age, gender, race and ethnicity, BMI, and health conditions including diabetes, hypertension, and CVD utilizing the 2016-2020 Medical Expenditure Panel Survey (MEPS). The DOC-M model employed a two-stage estimation process: a logit model to predict the probability of healthcare expenditures, followed by a generalized linear model with a log link and gamma distribution to ensure nationally representative cost estimations by incorporating MEPS design and weights.[21] This approach allowed us to calculate the expected annual healthcare spending per individual, reflecting demographic and health changes.

In our model, productivity costs were estimated to capture the economic impact of lost productivity due to morbidity and premature mortality associated with cardiovascular conditions, such as coronary heart disease (CHD) and stroke, as well as obesity and diabetes. We based our estimates on national data for productivity costs in 2021, dividing the total annual national productivity costs of each condition (CHD, stroke, CVD, diabetes, obesity) by the number of projected U.S. cases for each condition in the same

© 2025 Hwang JH et al. *JAMA Health Forum.*

year.[32-34] All costs were adjusted to 2023 dollars using the health care component of the Personal Consumption Expenditures price index.[35]

1.9 Validation of Outcomes

The DOC-M was validated by comparing the model output of relative percent weight change from baseline with reported intention-to-treat values. Additionally, the model was validated for changes in six cardiometabolic risk factors, including SBP and DBP, fasting blood glucose, total cholesterol, HDL-C, and triglycerides. This comparison ensured that the model accurately reflected the expected outcomes based on clinical trial data and real-world evidence. The validation process provided confidence in the model's ability to project long-term health outcomes and economic implications accurately.

1.10 Statistical Analysis

The DOC-M model is constructed to address various forms of uncertainty: stochastic, which captures the random variability in individual outcomes; parameter, dealing with the precision of model inputs; sampling, reflecting the representativeness of the cohort; and imputation, compensating for any missing data.[21] To mitigate stochastic uncertainty, the model can simulate an individual many times and take the average of these simulations. It also accounts for parameter uncertainty by integrating a variety of input parameters across numerous simulations. For example, a ten-fold replication of individuals, coupled with a thousand Monte Carlo simulations, leads to a significant number of total runs. The model also adjusts for sampling variation by using weighted averages to better estimate population outcomes. A modified version of Rubin's rule is employed to combine variance within and between simulations, providing a comprehensive measure of uncertainty in the results.[36,37] We estimated 95% uncertainty intervals for model outcomes.

To address the inherent uncertainty in our study, we conducted both scenario analyses and probabilistic sensitivity analysis (PSA).[38] In the scenario analysis conducted, each parameter was adjusted within its defined acceptable range at a time. These ranges were established based on available literature, ensuring that the variations applied in the analysis reflect real-world scenarios and outcomes.[30,39,40] This included evaluating different annual discount rates to consider the temporal valuation of costs and benefits, and assessing the ramifications of varied treatment discontinuation rates to reflect real-world patient adherence. In addition, we conducted a threshold analysis for AOM prices, establishing benchmarks at which the interventions align with ICER thresholds of $100000, $150000, $200000, $250000, and $300000 per QALY. These analyses structured to examine the cost-effectiveness of AOMs against different parameters critical to clinical and policy decision-making.

For our PSA, we performed 1000 Monte Carlo simulations to evaluate the uncertainty of our input variables. The choice of distributions for each parameter was based on the available data—beta distributions for health states and specific procedures, deterministic for the pricing of AOMs, normal for the AOM treatment effects, uniform for adverse effects, and gamma for healthcare costs. These distributions were adjusted to be representative of the US population and to encapsulate the uncertainties inherent in both the empirical data and the model's underlying assumptions.

© 2025 Hwang JH et al. *JAMA Health Forum.*

eTable 1. Percentages of Missing Values for Individual-Level Characteristics in the Model Population

Key Variable	Missing Values	Percentage Missing (%)
**Age**	0	0.0
**Sex**	0	0.0
**Race**	0	0.0
**Height**	692	8.1
**Weight**	685	8.0
**Body mass index**	699	8.2
**Systolic blood pressure**	1363	15.9
**Diastolic blood pressure**	1363	15.9
**Fasting glucose**	4798	56.1
**Glycated hemoglobin (HbA1c)**	1015	11.9
**Total cholesterol**	1162	13.6
**Low-density lipoprotein**	4895	57.3
**High-density lipoprotein**	1162	13.6
**Triglycerides**	4863	56.9
**History of smoking**	5	0.1
**History of diabetes**	3	0.04
**History of cardiovascular disease**	217	2.5
**History of hyperlipidemia**	1962	22.9
**History of hypertension**	11	0.1

© 2025 Hwang JH et al. *JAMA Health Forum.*

eTable 2. Key Model Inputs

Parameter	Mean	Variability	Distribution	Source
**Mean percent weight loss from baseline for non-diabetes, %**				
Lifestyle modification	−3.1	0.6[a]	Normal	Jastreboff et al. (2022)[8]
Naltrexone/bupropion	−6.1	0.30[a]	Normal	Greenway et al. (2010)[6]
Phentermine/topiramate	−9.8	0.43[a]	Normal	Gadde et al. (2011)[7]
Semaglutide	−14.9	3.70[a]	Normal	Wilding et al. (2021)[9]
Tirzepatide	−20.9	0.48[a]	Normal	Jastreboff et al. (2022)[8]
**Mean percent weight loss from baseline for diabetes, %**				
Lifestyle modification	−3.2	0.50[a]	Normal	Garvey et al. (2023)[11]
Naltrexone/bupropion	−5.0	0.30[a]	Normal	Hollander et al. (2013)[12]
Phentermine/topiramate	−8.8	0.60[a]	Normal	Gadde et al. (2011)[7], Garvey et al. (2014)[14]
Semaglutide	−9.7	0.40[a]	Normal	Davies et al. (2021)[13]
Tirzepatide	−14.7	0.50[a]	Normal	Garvey et al. (2023)[11]
**Treatment discontinuation, %**				
Lifestyle modification	2.6%	NA	Uniform	Jastreboff et al. (2022)[8]
Naltrexone/bupropion	19.5%	NA	Uniform	Greenway et al. (2010)[6]
Phentermine/topiramate	19%	NA	Uniform	Gadde et al. (2011)[7]
Semaglutide	7%	NA	Uniform	Wilding et al. (2021)[9]
Tirzepatide	6.2%	NA	Uniform	Jastreboff et al. (2022)[8]
**AOM-related AEs, %**				
**Naltrexone/bupropion**				
Nausea	29.8%	NA	Uniform	Greenway et al. (2010)[6]
Headache	13.8%	NA	Uniform	Greenway et al. (2010)[6]
Constipation	15.7%	NA	Uniform	Greenway et al. (2010)[6]
**Phentermine/topiramate**				
Paresthesia	21%	NA	Uniform	Gadde et al. (2011)[7]
Dry Mouth	21%	NA	Uniform	Gadde et al. (2011)[7]
Constipation	17%	NA	Uniform	Gadde et al. (2011)[7]

© 2025 Hwang JH et al. *JAMA Health Forum.*

Parameter	Mean	Variability	Distribution	Source
**Semaglutide**				
Nausea	44.2%	NA	Uniform	Wilding et al. (2021)[9]
Vomiting	24.8%	NA	Uniform	Wilding et al. (2021)[9]
Diarrhea	31.5%	NA	Uniform	Wilding et al. (2021)[9]
**Tirzepatide**				
Nausea	31%	NA	Uniform	Jastreboff et al. (2022)[8]
Vomiting	12.2%	NA	Uniform	Jastreboff et al. (2022)[8]
Diarrhea	23%	NA	Uniform	Jastreboff et al. (2022)[8]
**Utilities**				
Individual HRQOL	HRQOL prediction model	NA	Normal	Lubetkin et al. (2005)[20]
Coronary Heart Disease	−0.055	0.011	Beta	Davies et al. (2015)[41]
Stroke	−0.3	0.060	Beta	Davies et al. (2015)[41]
Nausea	−0.02	0.010[a]	Beta	Pollack et al. (2010)[26]
Vomiting	−0.02	0.010[a]	Beta	Pollack et al. (2010)[26]
Diarrhea	−0.02	0.010[a]	Beta	Pollack et al. (2010)[26]
Paresthesia	−0.012	0.004[a]	Beta	Matza et al. (2019)[25]
Dry Mouth	−0.011	0.004[a]	Beta	Matza et al. (2019)[25]
Constipation	−0.03	0.005[a]	Beta	Matza et al. (2019)[25]
Headache	−0.034	0.013[a]	Beta	Domitrz and Golicki (2022)[22]
**Disease-specific incidence and mortality**[21]				
Developing type 2 diabetes	Framingham Offspring Study 8-year diabetes risk model		Deterministic[b]	Wilson et al (2007)[2]
First ASCVD	ACC/AHA 10-year ASCVD risk model		Deterministic[b]	Goff et al (2014)[3]
% CHD vs. Stroke	Sex-race-specific values (47.4-64.0% vs. 36.0-52.6%)]		NA	Benjamin et al (2018)[42]
Second ASCVD	Framingham Heart Study 2-year risk model for second CHD		Deterministic[b]	D'Agostino et al (2000)[4]

© 2025 Hwang JH et al. *JAMA Health Forum.*

Parameter	Mean	Variability	Distribution	Source
% CHD vs. Stroke	Sex-race-specific values (58.0-73.2% vs. 26.8-42.0%)		NA	Benjamin et al (2018)[42]
Disease-specific mortality	CDC Wonder cause-specific mortality by age, sex, race/ethnicity groups		Beta	CDC (2018)[43]
**Probabilities among individuals undergoing RVSC[21]**				
% receiving RVSC	67.3%	0.2	Normal[b]	Lubetkin et al (2005)[20]
% CABG vs. PCI among RVSC	28.9 vs. 71.1%	0.2	Beta	Davies et al (2015)[41]
Death from CABG vs. PCI	1.8% vs. 2.1%	0.2	Beta	Davies et al (2015)[41]
**Costs, 2023 US $[c]**				
**Annual treatment costs**				
Lifestyle modification	1692	NA	NA	Zhang et al. (2020)[28]
Naltrexone/bupropion	420	NA	NA	FSS database[44]
Phentermine/topiramate	1786	NA	NA	FSS database[44]
Semaglutide	8412	NA	NA	Ippolito et al[30]
Tirzepatide	6236	NA	NA	Ippolito et al[30]
**Societal costs: average annual productivity loss per condition**				
CHD	7742	0.2	Gamma	Nelson et al[32]
CVD	6983	0.2	Gamma	Nelson et al[32]
Diabetes	818	0.2	Gamma	Park et al[34]
Obesity	679	0.2	Gamma	Cawley et al[33]
Stroke	5281	0.2	Gamma	Nelson et al[32]
**Healthcare costs[21]**				
Annual health care cost	Health care cost prediction model	NA	Normal	Kim et al. (2023)[21]
**Event-specific costs**				
CHD	11369	0.2	Gamma	CMS (2019)[45]

© 2025 Hwang JH et al. *JAMA Health Forum.*

Parameter	Mean	Variability	Distribution	Source
Stroke	18122	0.2	Gamma	CMS (2019)[45]
CABG	50464	0.2	Gamma	CMS (2019)[45]
PCI	20935	0.2	Gamma	CMS (2019)[45]

[a] Standard error manually derived from standard deviation or 95% confidence intervals.

[b] The distribution was based on regression coefficients.

[c] The cost of lifestyle modification was initially derived from the 2012 Look Ahead trial and adjusted for inflation to reflect 2023 U.S. dollars. Pricing for anti-obesity medications is based on estimated net prices in 2023 for naltrexone 32 mg combined with bupropion 360 mg, phentermine 15 mg plus topiramate 92 mg, semaglutide 2.4 mg, and tirzepatide 15 mg.

Abbreviations: AE, adverse events; AOM, anti-obesity medications; ASCVD, atherosclerotic cardiovascular disease; CABG, coronary artery bypass surgery, CHD, coronary heart disease; CVD, cardiovascular disease; HRQOL, health-related quality of life; NA, not applicable; PCI, percutaneous coronary intervention; RVSC, revascularization; SE, standard error.

© 2025 Hwang JH et al. *JAMA Health Forum.*

eTable 3. Treatment Efficacy of Lifestyle Modifications and Anti-Obesity Medications

Study Arms	Lifestyle modification	Naltrexone 32 mg-bupropion	Naltrexone 32 mg-bupropion	Phentermine 15mg-Topiramate 92mg	Phentermine 15mg-Topiramate 92mg	Semaglutide 2.4mg	Semaglutide 2.4mg	Tirzepatide 15mg	Tirzepatide 15mg
**Clinical Trial**	**SURMOUNT-1[8]**	**COR-I[6]**	**COR-DM[12]**	**CONQUER[7]**	**CONQUER-DM, OB-202/DM-230[7,14]**	**STEP-1[9]**	**STEP-2[13]**	**SURMOUNT-1[8]**	**SURMOUNT-2[11]**
**Mean percent weight loss, %**	−3.1 (0.6)	−6.1 (0.3)	−5.0 (0.3)	−9.8 (0.4)	−8.8 (0.6)	−14.9 (3.7)	−9.7 (0.4)	−20.9 (0.5)	−14.7 (0.5)
**SBP, mmHg**	−1.2 (0.5)	−0.1 (0.05)	0.0 (0.04)	–5·6 (0.5)	–4.2 (1.0)	–6.2 (1.5)	−3.9 (0.7)	–7.6 (0.5)	−7.7 (0.2)[a]
**DBP, mmHg**	−1.0 (0.4)	0.0 (0.3)	−1.1 (0.03)	–3·8 (0.3)	–2.4 (0.6)	–2.8 (0.2)[a]	−1.6 (0.4)	–4.6 (0.5)	−2.9 (0.2)[a]
**Fasting glucose, mg/dL**	0.9 (0.5)	−2.6 (0.4)	−11.9 (0.4)	−1.3 (0.6)	−11.9 (0.2)[a14]	–8.4 (0.2)[a]	−37.8 (1.8)	−10.6 (0.5)	−48.9 (2.3)
**Total cholesterol, % change in mg/dL**	−1.1 (0.7)	−1.4 (0.2)[a,b]	−0.9 (0.2)[a,b]	–6·3 (0.5)	–3.8 (1.4)	−3.0 (0.8)	−1.0 (0.2)[a]	–7.4 (6.1)	−2.2 (1.1)
**HDL, % change in mg/dL**	0.2 (0.7)	8.0 (0.9)	3.0 (0.03)	6·8 (0.7)	6·2 (1.4)	5.0 (0.8)	7.0 (0.2)[a]	8.2 (0.8)	9.6 (1.1)
**Triglycerides, % change in mg/dL**	−6.3 (1.5)	−12.7 (1.6)	−11.2 (2.3)	−10·6 (1.7)	−10·6 (5.6)	−22.0 (1.5)	−22.0 (0.2)[a]	–31.4 (1.07)	−30.6 (1.7)

Data are mean and standard error.

© 2025 Hwang JH et al. *JAMA Health Forum.*

Standard error manually derived from standard deviation or 95% confidence intervals.

For cardiometabolic risk factors, efficacy estimand was used when dose-specific treatment effect was available.

[a] If measures of standard error were missing or could not be estimated from the original clinical trials, they were assumed to be 0.2.

[b] Percent change in total cholesterol was estimated.

Abbreviation: SBP, systolic blood pressure; DBP, diastolic blood pressure; HDL, high-density lipoprotein.

© 2025 Hwang JH et al. *JAMA Health Forum.*

eTable 4. Estimated Lifetime Health Outcomes of Anti-Obesity Medications for NHANES Participants, by BMI Categories

**Overweight with comorbidities**				
**Estimated cases averted per 100000 eligible population[a]**	**Lifestyle modification + Medication**			
	**Naltrexone/ bupropion**	**Phentermine/ topiramate**	**Semaglutide**	**Tirzepatide**
	**Mean (95% UI)**	**Mean (95% UI)**	**Mean (95% UI)**	**Mean (95% UI)**
**Obesity[b]**	0 (0-0)	0 (0-0)	0 (0-0)	0 (0-0)
**Diabetes**	9498 (7518-11477)	5136 (4094-6178)	15861 (13267-18455)	15503 (12545-18461)
**CVD**	2137 (1656-2617)	3081 (2821-3341)	6822 (5870-7773)	8725 (7303-10147)
**First CVD**	981 (946-1016)	2779 (2773-2786)	4753 (4630-4876)	6258 (6058-6458)
**Second CVD**	2051 (1977-2125)	4142 (3956-4328)	5986 (5641-6332)	8360 (7939-8780)
**DM death**	584 (531-636)	454 (410-498)	1072 (959-1184)	1131 (1013-1248)
**CVD death**	212 (207-218)	1130 (1113-1147)	1778 (1689-1867)	2511 (2441-2581)
**Life years gained**	10908 (8332-13484)	21331 (18553-24109)	29054 (27392-30717)	42475 (36375-48575)
**Obesity I**				
**Estimated cases averted per 100000 eligible population[a]**	**Lifestyle modification + Medication**			
	**Naltrexone/ bupropion**	**Phentermine/ topiramate**	**Semaglutide**	**Tirzepatide**
	**Mean (95% UI)**	**Mean (95% UI)**	**Mean (95% UI)**	**Mean (95% UI)**
**Obesity**	15262 (13869-16654)	40384 (38558-42209)	67568 (65560-69576)	76315 (72437-80194)
**Diabetes**	11728 (10884-12572)	11670 (10741-12599)	19784 (17913-21655)	20913 (19218-22608)
**CVD**	3173 (3095-3251)	5717 (5672-5763)	9616 (9210-10022)	10754 (10192-11317)

© 2025 Hwang JH et al. *JAMA Health Forum.*

First CVD	1027 (986-1068)	3228 (3196-3260)	5575 (5563-5587)	7401 (7382-7421)
Second CVD	2498 (2338-2658)	5254 (4953-5555)	7184 (6792-7575)	10063 (9411-10714)
**DM death**	698 (649-746)	594 (565-622)	1394 (1295-1493)	1609 (1492-1726)
**CVD death**	312 (166-458)	1260 (1250-1270)	2085 (2045-2125)	2897 (2798-2995)
**Life years gained**	10464 (9769-11158)	20478 (18966-21990)	35520 (33566-37474)	48802 (47501-50104)
**Obesity II**				
**Estimated cases averted per 100000 eligible population**[a]	**Lifestyle modification + Medication**			
	**Naltrexone/ bupropion**	**Phentermine/ topiramate**	**Semaglutide**	**Tirzepatide**
	**Mean (95% UI)**	**Mean (95% UI)**	**Mean (95% UI)**	**Mean (95% UI)**
**Obesity**	0 (0-0)	0 (0-0)	16323 (11771-20876)	57384 (48030-66738)
**Diabetes**	10925 (8287-13564)	6123 (3959-8287)	17827 (14602-21052)	20742 (17037-24448)
**CVD**	1350 (1252-1448)	3793 (3352-4234)	6728 (5561-7896)	10397 (9269-11524)
First CVD	935 (919-951)	2726 (2705-2746)	5217 (4889-5545)	7538 (7237-7839)
Second CVD	2650 (2453-2847)	5210 (4930-5491)	7776 (7182-8369)	11247 (10290-12205)
**DM death**	616 (578-654)	234 (210-257)	1248 (1146-1350)	1680 (1524-1835)
**CVD death**	116 (50-183)	1131 (1122-1140)	1965 (1960-1970)	2925 (2902-2949)
**Life years gained**	11878 (11023-12733)	20877 (20117-21636)	40446 (39425-41467)	51495 (49885-53104)
**Obesity III**				
**Estimated cases averted per 100000 eligible population**[a]	**Lifestyle modification + Medication**			
	**Naltrexone/ bupropion**	**Phentermine/ topiramate**	**Semaglutide**	**Tirzepatide**
	**Mean**	**Mean**	**Mean**	**Mean**

© 2025 Hwang JH et al. *JAMA Health Forum.*

	(95% UI)	(95% UI)	(95% UI)	(95% UI)
**Obesity**	0 (0-0)	0 (0-0)	0 (0-0)	0 (0-0)
**Diabetes**	13175 (13037-13313)	5751 (5709-5793)	22277 (21043-23511)	24905 (22781-27029)
**CVD**	2708 (2638-2778)	4352 (4034-4670)	8449 (7915-8982)	12196 (11490-12902)
**First CVD**	947 (833-1061)	3026 (2913-3139)	6011 (5843-6178)	8347 (8237-8457)
**Second CVD**	2406 (2330-2482)	5133 (4701-5566)	7626 (7252-8001)	11004 (10296-11713)
**DM death**	696 (613-780)	364 (349-380)	1288 (1181-1396)	1507 (1366-1649)
**CVD death**	492 (457-527)	1158 (1075-1241)	2475 (2378-2572)	3393 (3268-3518)
**Life years gained**	13726 (13476-13975)	16893 (15827-17958)	39147 (40276-38018)	53355 (52194-54516)

Overweight was defined as a body mass index (BMI; calculated as weight in kilograms divided by height in meters squared) between 27 kg/m² and 29.9 kg/m². Obesity Class I was defined as a BMI between 30 kg/m² and 34.9 kg/m². Obesity Class II was defined as a BMI between 35 kg/m² and 39.9 kg/m². Obesity Class III was defined as a BMI of 40 kg/m² or higher.

[a] We calculated the difference in cumulative incidence of health outcomes between the lifestyle modification and anti-obesity medication interventions. This difference was multiplied by the total population size and then scaled to a per 100000 eligible population.

[b] Obesity is defined as BMI greater than or equal to 30 kg/m2.

Abbreviations: CVD, cardiovascular disease; DM, diabetes; NHANES, National Health and Nutrition Examination Survey; UI, uncertainty interval.

© 2025 Hwang JH et al. *JAMA Health Forum.*

eTable 5. Cost-Effectiveness Results of Lifestyle Modifications and Anti-Obesity Medications for NHANES Participants Over a Lifetime, by BMI Categories

**Overweight with comorbidities**					
	**Lifestyle modification**	**Lifestyle modification + Medication**			
		**Naltrexone/ bupropion**	**Phentermine/topiramate**	**Semaglutide**	**Tirzepatide**
	**Mean (95% UI)**	**Mean (95% UI)**	**Mean (95% UI)**	**Mean (95% UI)**	**Mean (95% UI)**
**Life years**	27.54 (26.31-28.77)	27.65 (26.39-28.90)	27.75 (26.49-29.01)	27.83 (26.58-29.08)	27.96 (26.67-29.26)
**Incremental life years**	[Reference]	0.11	0.21	0.29	0.42
**QALY**	15.28 (14.74-15.82)	15.33 (14.78-15.89)	15.43 (14.88-15.98)	15.48 (14.94-16.03)	15.57 (15.01-16.14)
**Incremental QALY**	[Reference]	0.05	0.15	0.20	0.29
**Total treatment cost, $**	29224	36124	56179	170107	135160
**Lifestyle modification cost, $**	29224 (28332-30117)	30010 (29090-30930)	30081 (29161-31001)	30119 (29212-31026)	30215 (29275-31155)
**Medication cost, $**	NA	6114 (5910-6318)	26097 (25223-26972)	139989 (135646-144331)	104944 (101607-108282)
**Health care expenditure, $**	166463 (160761-172164)	161448 (155488-167409)	157473 (151724-163222)	149713 (143654-155771)	144808 (139071-150545)
**Loss of productivity cost, $**	14689 (10848-18529)	12743 (8830-16657)	12743 (9469-16017)	10088 (6948-13228)	9082 (6402-11762)
**Total cost, $**	210376	210315	226394	329909	289049
**Incremental cost, $**	[Reference]	-61	16018	119533	78673
**ICER, $ per QALY gained**	[Reference]	Cost saving	106786	597665	271286
**Obesity I**					
		**Lifestyle modification + Medication**			

© 2025 Hwang JH et al. *JAMA Health Forum.*

	Lifestyle modification	Naltrexone/ bupropion	Phentermine/topiramate	Semaglutide	Tirzepatide
**Life years**	30.66 (29.80-31.51)	30.76 (29.90-31.62)	30.86 (29.99-31.73)	31.01 (30.14-31.89)	31.15 (30.28-32.01)
**Incremental life years**	[Reference]	0.10	0.20	0.36	0.49
**QALY**	16.55 (16.18-16.92)	16.61 (16.24-16.99)	16.72 (16.34-17.09)	16.81 (16.44-17.19)	16.91 (16.54-17.28)
**Incremental QALY**	[Reference]	0.06	0.17	0.26	0.36
**Total treatment cost, $**	31137	38492	59813	181274	144021
**Lifestyle modification cost, $**	31137 (30543-31732)	31994 (31389-32600)	32053 (31443-32663)	32136 (31528-32745)	32210 (31605-32815)
**Medication cost, $**	NA	6498 (6351-6644)	27760 (27149-28372)	149138 (146133-152142)	111811 (109600-114021)
**Health care expenditure, $**	168422 (162263-174580)	161934 (155587-168281)	156441 (150440-162443)	146539 (140686-152391)	139777 (133818-145736)
**Loss of productivity cost, $**	31405 (27690-35120)	25875 (22064-29686)	20321 (16555-24086)	12937 (9918-15956)	10265 (7489-13041)
**Total cost, $**	230964	226301	236575	340750	294063
**Incremental cost, $**	[Reference]	-4663	5611	109786	63099
**ICER, $ per QALY gained**	[Reference]	Cost saving	33005	422253	175275
**Obesity II**					
	**Lifestyle modification**	**Lifestyle modification + Medication**			
		**Naltrexone/ bupropion**	**Phentermine/topiramate**	**Semaglutide**	**Tirzepatide**
**Life years**	31.14 (29.84-32.45)	31.26 (29.95-32.58)	31.35 (30.04-32.67)	31.55 (30.23-32.87)	31.66 (30.33-32.98)
**Incremental life years**	[Reference]	0.12	0.21	0.40	0.51
**QALY**	16.51 (16.01-17.02)	16.57 (16.07-17.08)	16.67 (16.15-17.19)	16.81 (16.28-17.34)	16.89 (16.36-17.43)
**Incremental QALY**	[Reference]	0.06	0.15	0.29	0.38

© 2025 Hwang JH et al. *JAMA Health Forum.*

**Total treatment cost, $**	31392	38799	60371	183066	145276
**Lifestyle modification cost, $**	31392 (30569-32216)	32249 (31414-33083)	32310 (31474-33146)	32435 (31599-33271)	32477 (31637-33316)
**Medication cost, $**	NA	6550 (6348-6752)	28061 (27226-28897)	150631 (146573-154690)	112800 (109747-115852)
**Health care expenditure, $**	191848 (183813-199884)	185843 (177288-194399)	181742 (173492-189992)	169386 (160682-178089)	160543 (151604-169483)
**Loss of productivity cost, $**	34524 (31179-37869)	31877 (28899-34854)	31629 (28822-34435)	26091 (23156-29025)	16871 (13878-19865)
**Total cost, $**	257764	256519	273742	378543	322691
**Incremental cost, $**	[Reference]	-1245	15978	120779	64927
**ICER, $ per QALY gained**	[Reference]	Cost saving	106520	416479	170860
**Obesity III**					
	**Lifestyle modification**	**Lifestyle modification + Medication**			
		**Naltrexone/ bupropion**	**Phentermine/topiramate**	**Semaglutide**	**Tirzepatide**
**Life years**	33.26 (32.13-34.39)	33.40 (32.27-34.53)	33.43 (32.29-34.57)	33.65 (32.53-34.77)	33.79 (32.65-34.93)
**Incremental life years**	[Reference]	0.14	0.17	0.39	0.53
**QALY**	17.32 (16.88-17.76)	17.40 (16.96-17.84)	17.44 (16.99-17.89)	17.58 (17.14-18.02)	17.68 (17.23-18.12)
**Incremental QALY**	[Reference]	0.08	0.12	0.26	0.36
**Total treatment cost, $**	33005	40776	63328	192221	152640
**Lifestyle modification cost, $**	33005 (32231-33779)	33907 (33144-34670)	33930 (33155-34705)	34052 (33298-34806)	34137 (33376-34897)
**Medication cost, $**	NA	6869 (6684-7054)	29398 (28583-30212)	158169 (154440-161899)	118503 (115720-121286)
**Health care expenditure, $**	225938 (219386-232490)	217681 (211180-224182)	214771 (208670-220873)	201259 (194822-207696)	192467 (186327-198606)

© 2025 Hwang JH et al. *JAMA Health Forum.*

**Loss of productivity cost, $**	37010 (32972-41047)	34768 (30833-38703)	34987 (30930-39043)	30960 (27623-34298)	29395 (26282-32508)
**Total cost, $**	295953	293225	313086	424440	374502
**Incremental cost, $**	[Reference]	-2728	17133	128487	78549
**ICER, $ per QALY gained**	[Reference]	Cost saving	142775	494180	218191

Overweight was defined as a body mass index (BMI; calculated as weight in kilograms divided by height in meters squared) between 27 kg/m² and 29.9 kg/m². Obesity Class I was defined as a BMI between 30 kg/m² and 34.9 kg/m². Obesity Class II was defined as a BMI between 35 kg/m² and 39.9 kg/m². Obesity Class III was defined as a BMI of 40 kg/m² or higher.

ICERs were calculated as the estimated mean net change in costs from a health care perspective divided by the mean net change in QALYs. ICERs below $100000/QALY were considered cost effective.

Abbreviations: ICER, incremental cost-effectiveness ratio; NA, not applicable; NHANES, National Health and Nutrition Examination Survey; QALY, quality-adjusted life-year; UI, uncertainty interval.

© 2025 Hwang JH et al. *JAMA Health Forum.*

eTable 6. Cost-Effectiveness Results of Lifestyle Modifications and Anti-Obesity Medications Based on Comorbidities

	Lifestyle modification		Lifestyle modification + Medication							
			Naltrexone/ bupropion		Phentermine/ topiramate		Semaglutide		Tirzepatide	
	Without comorbidities	With comorbidities	Without comorbidities	With comorbidities	Without comorbidities	With comorbidities	Without comorbidities	With comorbidities	Without comorbidities	With comorbidities
**Number of eligible population**	756	4067	756	4067	756	4067	756	4067	756	4067
**Number of representative population**	18932830	107599881	18932830	107599881	18932830	107599881	18932830	107599881	18932830	107599881
	**Mean (95% UI)**	**Mean (95% UI)**	**Mean (95% UI)**	**Mean (95% UI)**	**Mean (95% UI)**	**Mean (95% UI)**	**Mean (95% UI)**	**Mean (95% UI)**	**Mean (95% UI)**	**Mean (95% UI)**
**Health outcome measures**										
**Life years**	39.70 (38.60-40.80)	28.84 (28.01-29.66)	39.97 (38.90-41.03)	28.95 (28.16-29.75)	39.97 (38.91-41.04)	29.06 (28.25-29.87)	40.05 (39.00-41.11)	29.17 (28.35-30.00)	40.25 (39.20-41.31)	29.35 (28.56-30.14)
**Incremental life years**	[Reference]	[Reference]	0.27	0.11	0.27	0.22	0.35	0.33	0.55	0.51
**QALY**	20.64 (20.27-21.01)	15.62 (15.27-15.97)	20.76 (20.40-21.12)	15.69 (15.35-16.03)	20.78 (20.42-21.14)	15.79 (15.44-16.14)	20.82 (20.46-21.17)	15.88 (15.53-16.24)	20.91 (20.55-21.27)	15.99 (15.65-16.33)
**Incremental QALY**	[Reference]	[Reference]	0.12	0.07	0.14	0.17	0.18	0.26	0.27	0.37
**Costs, $**										

© 2025 Hwang JH et al. *JAMA Health Forum.*

	Lifestyle modification		Lifestyle modification + Medication							
			Naltrexone/ bupropion		Phentermine/ topiramate		Semaglutide		Tirzepatide	
**Total treatment cost, $**	36926	30023	45622	37166	70844	57855	214341	175111	174201	139082
**Lifestyle modification cost, $**	36926 (36312-37541)	30023 (29445-30601)	37929 (37341-38518)	30871 (30298-31444)	37939 (37359-38518)	30929 (30344-31514)	37943 (37363-38522)	30991 (30404-31578)	38056 (37482-38629)	31095 (30530-31660)
**Medication cost, $**	NA	NA	7693 (7525-7860)	6295 (6170-6420)	32905 (32137-33674)	26926 (26411-27442)	176398 (173311-179485)	144120 (141470-146771)	136145 (134087-138202)	107987 (106018-109956)
**Health care expenditure, $**	118608 (113931-123284)	193192 (187744-198640)	111032 (106563-115500)	187195 (181699-192691)	107575 (103160-111990)	182533 (177114-187952)	97823 (93266-102380)	171340 (165990-176690)	91597 (87137-96057)	164427 (159262-169593)
**Loss of productivity cost, $**	29165 (27099-31230)	32006 (28949-35063)	25017 (23124-26910)	29826 (26700-32952)	20386 (18193-22580)	26948 (24102-29795)	15400 (13444-17357)	21468 (19253-23684)	11802 (10193-13412)	17479 (15336-19623)
**Total cost, $**	181462	223703	177551	219677	200345	242505	363629	397622	302844	327454
**Incremental cost, $**	[Reference]	[Reference]	-3911	-4026	18883	18802	182167	173919	121382	103751
**ICER, $ per QALY gained**	[Reference]	[Reference]	Cost saving	Cost saving	134879	110600	1012039	668919	449563	280408

Presence of comorbidities is defined as having one or more of the following conditions: diabetes, hypertension, dyslipidemia, or cardiovascular disease (CVD).

ICERs were calculated as the estimated mean net change in costs from a health care perspective divided by the mean net change in QALYs. ICERs below $100000/QALY were considered cost effective.

© 2025 Hwang JH et al. *JAMA Health Forum.*

Abbreviations: ICER, incremental cost-effectiveness ratio; NA, not applicable; QALY, quality-adjusted life-year; UI, uncertainty interval.

© 2025 Hwang JH et al. *JAMA Health Forum.*

eTable 7. Cost-Effectiveness Results of Lifestyle Modifications and Anti-Obesity Medications Based on Diabetes Status

	Lifestyle modification		Lifestyle modification + Medication							
			Naltrexone/ bupropion		Phentermine/ topiramate		Semaglutide		Tirzepatide	
	Without diabetes	With diabetes	Without diabetes	With diabetes	Without diabetes	With diabetes	Without diabetes	With diabetes	Without diabetes	With diabetes
**Number of eligible population**	3762	1061	3762	1061	3762	1061	3762	1061	3762	1061
**Number of representative population**	104844837	21687875	104844837	21687875	104844837	21687875	104844837	21687875	104844837	21687875
	**Mean (95% UI)**	**Mean (95% UI)**	**Mean (95% UI)**	**Mean (95% UI)**	**Mean (95% UI)**	**Mean (95% UI)**	**Mean (95% UI)**	**Mean (95% UI)**	**Mean (95% UI)**	**Mean (95% UI)**
**Health outcome measures**										
**Life years**	32.34 (31.58-33.09)	21.40 (20.11-22.69)	32.41 (31.70-33.12)	21.46 (20.18-22.73)	32.49 (31.77-33.21)	21.54 (20.22-22.87)	32.73 (31.96-33.50)	21.60 (20.22-22.97)	32.85 (32.12-33.57)	21.76 (20.36-23.17)
**Incremental life years**	[Reference]	[Reference]	0.07	0.06	0.15	0.14	0.39	0.20	0.51	0.36
**QALY**	17.26 (16.94-17.58)	12.04 (11.48-12.61)	17.32 (17.03-17.61)	12.06 (11.50-12.62)	17.38 (17.09-17.68)	12.12 (11.54-12.70)	17.55 (17.24-17.86)	12.14 (11.54-12.73)	17.63 (17.34-17.93)	12.23 (11.62-12.84)
**Incremental QALY**	[Reference]	[Reference]	0.06	0.02	0.12	0.08	0.29	0.10	0.37	0.19
**Costs, $**										
**Total treatment cost, $**	31486	24973	38435	30867	55664	48056	178057	145038	142671	115786

© 2025 Hwang JH et al. *JAMA Health Forum.*

	Lifestyle modification		Lifestyle modification + Medication							
			Naltrexone/ bupropion		Phentermine/ topiramate		Semaglutide		Tirzepatide	
	Without diabetes	With diabetes	Without diabetes	With diabetes	Without diabetes	With diabetes	Without diabetes	With diabetes	Without diabetes	With diabetes
**Lifestyle modification cost, $**	31486 (30957-32015)	24973 (23957-25990)	33187 (32724-33650)	25622 (24620-26625)	33209 (32727-33692)	25689 (24637-26741)	33354 (32850-33858)	25733 (24643-26822)	33418 (32932-33904)	25863 (24760-26966)
**Medication cost, $**	NA	NA	5248 (5169-5327)	5245 (5024-5466)	22455 (22001-22909)	22367 (21405-23329)	144703 (142402-147004)	119305 (114467-124143)	109253 (107636-110870)	89923 (86141-93704)
**Health care expenditure, $**	166429 (161616-171241)	256156 (247366-264946)	161614 (156439-166789)	255540 (247642-263438)	157876 (152935-162816)	252889 (243768-262010)	143737 (138872-148601)	251854 (242925-260783)	136287 (131749-140824)	248356 (240011-256700)
**Loss of productivity cost, $**	30670 (28332-33008)	63871 (50399-77343)	27544 (25125-29963)	59538 (51892-67183)	25734 (23310-28159)	56734 (45179-68288)	19199 (17463-20936)	56403 (45654-67151)	16080 (14311-17850)	53652 (45157-62147)
**Total cost, $**	206454	419285	201460	417499	221148	441209	370768	610950	306449	559098
**Incremental cost, $**	[Reference]	[Reference]	-4994	-1789	14694	21924	164314	191665	99995	139813
**ICER, $ per QALY gained**	[Reference]	[Reference]	Cost saving	Cost saving	122450	274050	566600	1916650	270257	735858

ICERs were calculated as the estimated mean net change in costs from a health care perspective divided by the mean net change in QALYs. ICERs below $100000/QALY were considered cost effective.

Abbreviations: ICER, incremental cost-effectiveness ratio; NA, not applicable; QALY, quality-adjusted life-year.

© 2025 Hwang JH et al. *JAMA Health Forum.*

eTable 8. Cost-Effectiveness Results of Lifestyle Modifications and Anti-Obesity Medications Based on Age Groups

	Lifestyle modification			Lifestyle modification + Medication											
				Naltrexone/ bupropion			Phentermine/ topiramate			Semaglutide			Tirzepatide		
	**20-39**	**40-64**	**≥65**	**20-39**	**40-64**	**≥65**	**20-39**	**40-64**	**≥65**	**20-39**	**40-64**	**≥65**	**20-39**	**40-64**	**≥65**
**No. of eligible population**	13440	24160	10630	13440	24160	10630	13440	24160	10630	13440	24160	10630	13440	24160	10630
**Number of representative population**	41054326	61283888	24194496	41054326	61283888	24194496	41054326	61283888	24194496	41054326	61283888	24194496	41054326	61283888	24194496
**Health outcome measures**															
**Life years**	46.16	26.93	12.95	46.31	27	12.96	46.35	27.05	12.97	46.53	27.26	13.12	46.75	27.48	13.11
**Incremental life years**	[Reference]	[Reference]	[Reference]	0.15	0.07	0.01	0.19	0.12	0.02	0.37	0.33	0.17	0.59	0.55	0.16
**QALY**	22.58	15.25	8.71	22.67	15.29	8.72	22.73	15.38	8.75	22.87	15.51	8.83	22.99	15.64	8.86
**Incremental QALY**	[Reference]	[Reference]	[Reference]	0.09	0.04	0.01	0.15	0.13	0.04	0.29	0.26	0.12	0.41	0.39	0.15
**Costs, $**															
**Total treatm**	40563	29817	18192	50070	36835	22430	77730	57237	34922	235802	174217	105930	187213	138529	83915

© 2025 Hwang JH et al. *JAMA Health Forum.*

	Lifestyle modification			Lifestyle modification + Medication											
				Naltrexone/ bupropion			Phentermine/ topiramate			Semaglutide			Tirzepatide		
	20-39	40-64	≥65	20-39	40-64	≥65	20-39	40-64	≥65	20-39	40-64	≥65	20-39	40-64	≥65
**ent cost, $**															
**Lifestyle modification cost, $**	40563	29817	18192	41649	30612	18615	41658	30652	18606	41753	143422	18755	41842	30943	18749
**Medication cost, $**	NA	NA	NA	8421	6223	3815	36072	26585	16316	194049	30795	87175	145371	107586	65166
**Health care expenditure, $**	145787	208005	184516	134887	203155	183084	130248	198022	179716	114549	187832	176975	105167	181250	173255
**Loss of productivity cost, $**	18752	27842	20273	16551	26703	19768	15375	24779	18493	12216	20322	16858	9792	18283	16431
**Total cost, $**	205102	265664	222981	201508	266693	225282	223353	280038	233131	362567	382371	299763	302172	338062	273601
**Incremental cost, $**	[Reference]	[Reference]	[Reference]	-3595	1030	2301	18250	14375	10150	157464	116708	76782	97070	72399	50620
**ICER, $ per QALY gained**	[Reference]	[Reference]	[Reference]	Cost saving	25743	230099	121669	110576	253749	542981	448877	639850	236756	185638	337467

© 2025 Hwang JH et al. *JAMA Health Forum.*

ICERs were calculated as the estimated mean net change in costs from a health care perspective divided by the mean net change in QALYs. ICERs below $100000/QALY were considered cost effective.

Abbreviations: ICER, incremental cost-effectiveness ratio; NA, not applicable; QALY, quality-adjusted life-year.

© 2025 Hwang JH et al. *JAMA Health Forum.*

eTable 9. Cost-Effectiveness Results of No intervention Vs. Anti-Obesity Medications

	No intervention	Lifestyle modification + Medication			
		Naltrexone/ bupropion	Phentermine/ topiramate	Semaglutide	Tirzepatide
	Mean (95% UI)	Mean (95% UI)	Mean (95% UI)	Mean (95% UI)	Mean (95% UI)
**Health outcome measures**					
**Life years**	30.45 (29.71-31.19)	30.58 (29.82-31.35)	30.67 (29.90-31.43)	30.82 (30.05-31.60)	30.97 (30.20-31.74)
**Incremental life years**	[Reference]	0.11	0.20	0.35	0.50
**QALY**	16.38 (16.07-16.69)	16.44 (16.12-16.76)	16.52 (16.20-16.85)	16.63 (16.30-16.96)	16.73 (16.41-17.05)
**Incremental QALY**	[Reference]	0.06	0.14	0.25	0.35
**Costs, $**					
**Total treatment cost, $**	NA	38392	59705	180936	143744
**Lifestyle modification cost, $**	NA	31894 (31361-32426)	31954 (31419-32490)	32039 (31496-32582)	32134 (31599-32670)
**Medication cost, $**	NA	6498 (6379-6616)	27751 (27249-28254)	148897 (146318-151476)	111610 (109738-113482)
**Health care expenditure, $**	190205 (183968-196442)	175947 (170838-181055)	171637 (166511-176764)	160974 (156125-165823)	154028 (149226-158829)
**Loss of productivity cost, $**	34483 (32435-36531)	27211 (24936-29486)	24921 (22601-27241)	19340 (17443-21238)	15517 (13774-17260)
**Total cost, $**	224688	241550	256263	361250	313289
**Incremental cost, $**	[Reference]	16862	31575	136562	88601

© 2025 Hwang JH et al. *JAMA Health Forum.*

	No intervention	Lifestyle modification + Medication			
		**Naltrexone/ bupropion**	**Phentermine/ topiramate**	**Semaglutide**	**Tirzepatide**
	**Mean (95% UI)**	**Mean (95% UI)**	**Mean (95% UI)**	**Mean (95% UI)**	**Mean (95% UI)**
**ICER, $ per QALY gained[c]**	[Reference]	240886	210500	525238	246114

ICERs were calculated as the estimated mean net change in costs from a health care perspective divided by the mean net change in QALYs. ICERs below $100000/QALY were considered cost effective.

Abbreviations: ICER, incremental cost-effectiveness ratio; NA, not applicable; QALY, quality-adjusted life-year; UI, uncertainty interval.

© 2025 Hwang JH et al. *JAMA Health Forum.*

eTable 10. Cost-Effectiveness Results of Lifestyle Modifications and Anti-Obesity Medications Varying Treatment Discontinuation Rate

**Naltrexone/bupropion**							
**Discontinuation rate, %**	**5**	**10**	**Base Case**	**20**	**30**	**40**	**50**
**Life years**	30.62	30.61	30.58	30.59	30.57	30.55	30.51
**Incremental life years**	0.15	0.14	0.11	0.12	0.10	0.08	0.04
**QALY**	16.46	16.45	16.44	16.44	16.43	16.41	16.39
**Incremental QALY**	0.08	0.08	0.06	0.058	0.05	0.04	0.01
**Total treatment cost, $**	39489	39112	38389	38344	37580	36822	36109
**Lifestyle modification cost, $**	31922	31920	31908	31904	31895	31886	31857
**Medication cost, $**	7568	7192	6482	6440	5685	4936	4252
**Health care expenditure, $**	174342	174932	176102	176099	177362	178468	179492
**Loss of productivity cost, $**	25856	26113	27211	27512	27827	28417	29289
**Total cost, $**	239688	240157	241703	241955	242769	243707	244890
**Incremental cost, $**	-4643	-4174	-2628	-2376	-1562	-624	559
**ICER, $, QALY gained**	Cost saving	Cost saving	Cost saving	Cost saving	Cost saving	Cost saving	55900
**Phentermine/topiramate**							
**Discontinuation rate, %**	**5**	**10**	**Base Case**	**20**	**30**	**40**	**50**
**Life years**	30.72	30.71	30.67	30.64	30.63	30.62	30.56

© 2025 Hwang JH et al. *JAMA Health Forum.*

**Incremental life years**	0.25	0.24	0.20	0.17	0.16	0.15	0.09
**QALY**	16.56	16.55	16.53	16.52	16.50	16.48	16.45
**Incremental QALY**	0.18	0.18	0.15	0.14	0.12	0.11	0.07
**Total treatment cost, $**	64144	62561	59675	59295	56094	52902	50225
**Lifestyle modification cost, $**	31980	31985	31964	31940	31936	31935	31885
**Medication cost, $**	32165	30576	27711	27355	24159	20967	18340
**Health care expenditure, $**	169210	170241	171682	171744	173502	175470	176550
**Loss of productivity cost, $**	23498	24394	24921	24994	25396	26320	27904
**Total cost, $**	256853	257196	256278	256033	254993	254692	254679
**Incremental cost, $**	12522	12865	11947	11702	10662	10361	10348
**ICER, $, QALY gained**	69567	71472	79647	83586	88850	94191	147829
**Semaglutide**							
**Discontinuation rate, %**	**5**	**Base Case**	**10**	**20**	**30**	**40**	**50**
**Life years**	30.84	30.83	30.82	30.79	30.74	30.70	30.65
**Incremental life years**	0.37	0.36	0.35	0.31	0.27	0.23	0.18
**QALY**	16.64	16.63	16.62	16.60	16.56	16.53	16.49
**Incremental QALY**	0.26	0.26	0.24	0.22	0.18	0.15	0.11
**Total treatment cost, $**	183931	180891	176436	161387	146103	130999	115783

© 2025 Hwang JH et al. *JAMA Health Forum.*

**Lifestyle modification cost, $**	32055	32051	32039	32026	31999	31975	31942
**Medication cost, $**	151877	148840	144398	129361	114104	99025	83841
**Health care expenditure, $**	160561	161167	161901	164498	167182	169869	172532
**Loss of productivity cost, $**	18858	19340	19269	20717	22289	23226	24092
**Total cost, $**	363351	361398	357607	346602	335574	324095	312407
**Incremental cost, $**	119020	117067	113276	102271	91243	79764	68076
**ICER, $, QALY gained**	457769	450258	471983	464868	506906	531760	618873
**Tirzepatide**							
**Discontinuation rate, %**	**5**	**Base Case**	**10**	**20**	**30**	**40**	**50**
**Life years**	31.00	30.96	30.95	30.87	30.81	30.76	30.70
**Incremental life years**	0.53	0.49	0.47	0.40	0.34	0.29	0.23
**QALY**	16.74	16.72	16.71	16.67	16.62	16.58	16.54
**Incremental QALY**	0.37	0.35	0.33	0.29	0.24	0.21	0.16
**Total treatment cost, $**	145128	143671	139400	128171	116767	105538	94378
**Lifestyle modification cost, $**	32157	32126	32124	32076	32032	32015	31976
**Medication cost, $**	112971	111546	107277	96095	84735	73523	62403
**Health care expenditure, $**	153855	154071	155399	158559	162041	165494	168904
**Loss of productivity cost, $**	15375	15517	16042	17994	19616	21455	22475

© 2025 Hwang JH et al. *JAMA Health Forum.*

**Total cost, $**	314358	313260	310842	304724	298424	292487	285758
**Incremental cost, $**	70027	68929	66511	60393	54093	48156	41427
**ICER, $, QALY gained**	189262	196940	201548	208252	225388	229314	258919

Individuals who experienced adverse effects and met pre-defined discontinuation criteria were removed from the treatment group within the first year. Following discontinuation, these individuals were transitioned to lifestyle modification. If lifestyle modification was also discontinued, individuals received no further treatment. Participants who discontinued treatment within the first year had their weight and cardiovascular risk factors revert to baseline by the end of the second year. Subsequently, the effects of lifestyle modification or the natural progression of weight and cardiovascular risk factors were considered in the analysis.

ICERs were calculated as the estimated mean net change in costs from a health care perspective divided by the mean net change in QALYs. ICERs below $100000/QALY were considered cost effective.

Abbreviations: ICER, incremental cost-effectiveness ratio; QALY, quality-adjusted life-year.

© 2025 Hwang JH et al. *JAMA Health Forum.*

eTable 11. Cost-Effectiveness Results of Lifestyle Modifications and Anti-Obesity Medications with 10-year of Treatment Adherence

	**Lifestyle modification**	**Lifestyle modification + Medication**			
		**Naltrexone/ bupropion**	**Phentermine/ topiramate**	**Semaglutide**	**Tirzepatide**
	**Mean (95% UI)**	**Mean (95% UI)**	**Mean (95% UI)**	**Mean (95% UI)**	**Mean (95% UI)**
**Health outcome measures**					
**Life years**	30.38 (29.63-31.14)	30.54 (29.78-31.29)	30.67 (29.88-31.46)	30.87 (30.07-31.66)	30.99 (30.21-31.78)
**Incremental life years**	[Reference]	0.16	0.29	0.49	0.61
**QALY**	16.34 (16.02-16.66)	16.41 (16.09-16.73)	16.52 (16.18-16.85)	16.63 (16.30-16.97)	16.73 (16.40-17.06)
**Incremental QALY**	[Reference]	0.07	0.18	0.29	0.39
**Costs, $**					
**Total treatment cost, $**	14891	18048	28627	86336	68427
**Lifestyle modification cost, $**	14891 (14827-14955)	15224 (15161-15288)	15239 (15170-15307)	15241 (15173-15309)	15258 (15193-15322)
**Medication cost, $**	NA	2824 (2803-2845)	13388 (13285-13491)	71095 (70668-71523)	53169 (52876-53463)
**Health care expenditure, $**	183375 (178374-188376)	176607 (171275-181939)	172717 (167483-177950)	161600 (156675-166525)	155021 (150067-159976)
**Loss of productivity cost, $**	30456 (28188-32724)	26824 (24845-28803)	24697 (22624-26770)	19110 (17251-20968)	15817 (13836-17798)
**Total cost, $**	228722	221479	226041	267046	239265
**Incremental cost, $**	[Reference]	-7243	-2681	38324	10543
**ICER, $ per QALY gained[c]**	[Reference]	Cost saving	Cost saving	132152	27033

© 2025 Hwang JH et al. *JAMA Health Forum.*

ICERs were calculated as the estimated mean net change in costs from a health care perspective divided by the mean net change in QALYs. ICERs below $100000/QALY were considered cost effective.

Abbreviations: ICER, incremental cost-effectiveness ratio; NA, not applicable; QALY, quality-adjusted life-year; UI, uncertainty interval.

© 2025 Hwang JH et al. *JAMA Health Forum.*

eTable 12. Percentage of Simulations Achieving Cost-Effectiveness at Various Willingness-to-Pay Thresholds

Willingness-to-Pay Threshold ($/QALY), mean % (95% CI)	Naltrexone/bupropion	Phentermine/topiramate	Semaglutide	Tirzepatide
$100000 per QALY	89.1% (87.2-91.0)	23.5% (20.9-26.1)	0.0% (0.0–0.0)	0.0% (0.0–0.0)
$150000 per QALY	89.7% (87.8-91.6)	62.9% (59.9-65.9)	0.0% (0.0–0.0)	0.0% (0.0–0.0)
$200000 per QALY	91.2% (89.5-93.0)	83.4% (81.1-85.7)	0.0% (0.0–0.0)	0.0% (0.0–0.0)
$250000 per QALY	92.7% (91.1-94.3)	91.9% (90.2-93.6)	0.0% (0.0–0.0)	9.6% (7.8-11.4)
$300000 per QALY	93.7% (92.2-95.2)	95.8% (94.6-97.0)	0.0% (0.0–0.0)	49.3% (46.2-52.4)

The percentages reflect the proportion of probabilistic sensitivity analysis (PSA) simulations where each treatment achieved cost-effectiveness under the specified willingness-to-pay (WTP) thresholds per QALY. Values in parentheses indicate the 95% confidence intervals for each percentage.

© 2025 Hwang JH et al. *JAMA Health Forum.*

eTable 13. Cost-Effectiveness Results of Lifestyle Modifications and Anti-Obesity Medications Varying Annual Discount Rate

	Lifestyle modification			Lifestyle modification + Medication											
				Naltrexone/ bupropion			Phentermine/ topiramate			Semaglutide			Tirzepatide		
	**0%**	**1.5%**	**5%**	**0%**	**1.5%**	**5%**	**0%**	**1.5%**	**5%**	**0%**	**1.5%**	**5%**	**0%**	**1.5%**	**5%**
**Life years**	30.49	30.49	30.49	30.56	30.56	30.56	30.62	30.62	30.62	30.85	30.85	30.85	30.95	30.95	30.95
**Incremental life years**	[Reference]	[Reference]	[Reference]	0.07	0.07	0.07	0.13	0.13	0.13	0.36	0.36	0.36	0.46	0.46	0.46
**QALY**	26.29	20.26	12.95	26.43	20.38	12.99	26.57	20.52	13.04	26.90	20.70	13.12	27.06	20.83	13.18
**Incremental QALY**	[Reference]	[Reference]	[Reference]	0.13	0.12	0.03	0.27	0.26	0.09	0.60	0.44	0.16	0.77	0.57	0.23
**Total treatment cost, $**	51841	39305	23779	64054	48622	29372	99391	75711	45616	302966	230029	138156	240865	182830	109761
**Lifestyle modification cost, $**	51841	39305	23779	53284	40422	24398	53387	40565	24413	53770	40745	24474	53935	40884	24536
**Medication cost, $**	NA	NA	NA	10770	8200	4974	46004	35146	21203	249196	189284	113682	186930	141946	85225
**Health care expenditure, $**	296894	228129	144376	283664	219082	139816	277009	214352	136441	256562	199513	128448	244713	190882	123580

© 2025 Hwang JH et al. *JAMA Health Forum.*

	Lifestyle modification			Lifestyle modification + Medication											
				Naltrexone/ bupropion			Phentermine/ topiramate			Semaglutide			Tirzepatide		
	**0%**	**1.5%**	**5%**	**0%**	**1.5%**	**5%**	**0%**	**1.5%**	**5%**	**0%**	**1.5%**	**5%**	**0%**	**1.5%**	**5%**
**Loss of productivity cost, $**	82793	48252	18505	73575	42724	16245	68491	39574	14829	54040	30908	11360	45669	25855	9280
**Total cost, $**	431528	315686	186660	421293	310428	185433	444891	329637	196886	613568	460450	277964	531247	399567	242621
**Incremental cost, $**	[Reference]	[Reference]	[Reference]	-10235	-5258	-1227	13363	13951	10226	182040	144764	91304	99719	83881	55961
**ICER, $ per QALY gained**	[Reference]	[Reference]	[Reference]	Cost-saving	Cost-saving	Cost saving	49493	53658	113622	303400	329009	570650	129505	147160	243309

An annual discount rate is applied in this cost-effectiveness analysis to account for the time value of money and health benefits. ICERs were calculated as the estimated mean net change in costs from a health care perspective divided by the mean net change in QALYs. ICERs below $100000/QALY were considered cost effective.

Abbreviations: ICER, incremental cost-effectiveness ratio; NA, not applicable; QALY, quality-adjusted life-year.

© 2025 Hwang JH et al. *JAMA Health Forum.*

eFigure 1. Estimated Lifetime Health Outcomes of Anti-Obesity Medications for NHANES Participants

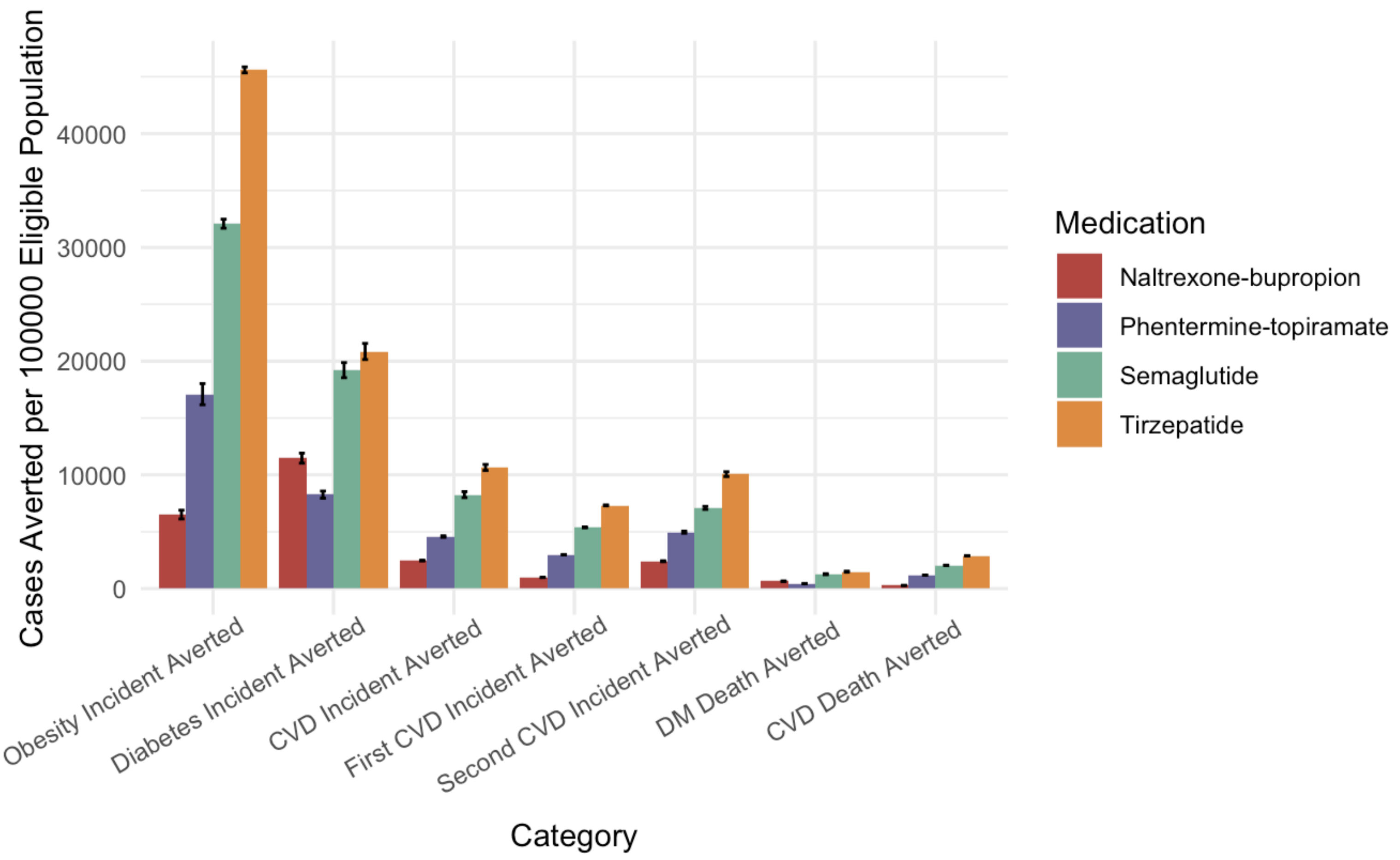

Error bars represent 95% uncertainty interval.
Abbreviations: CVD, cardiovascular disease; DM, diabetes; NHANES, National Health and Nutrition Examination Survey.

© 2025 Hwang JH et al. *JAMA Health Forum.*

eFigure 2. Threshold Analysis for Annual Net Cost of Tirzepatide and Semaglutide

A. Tirzepatide

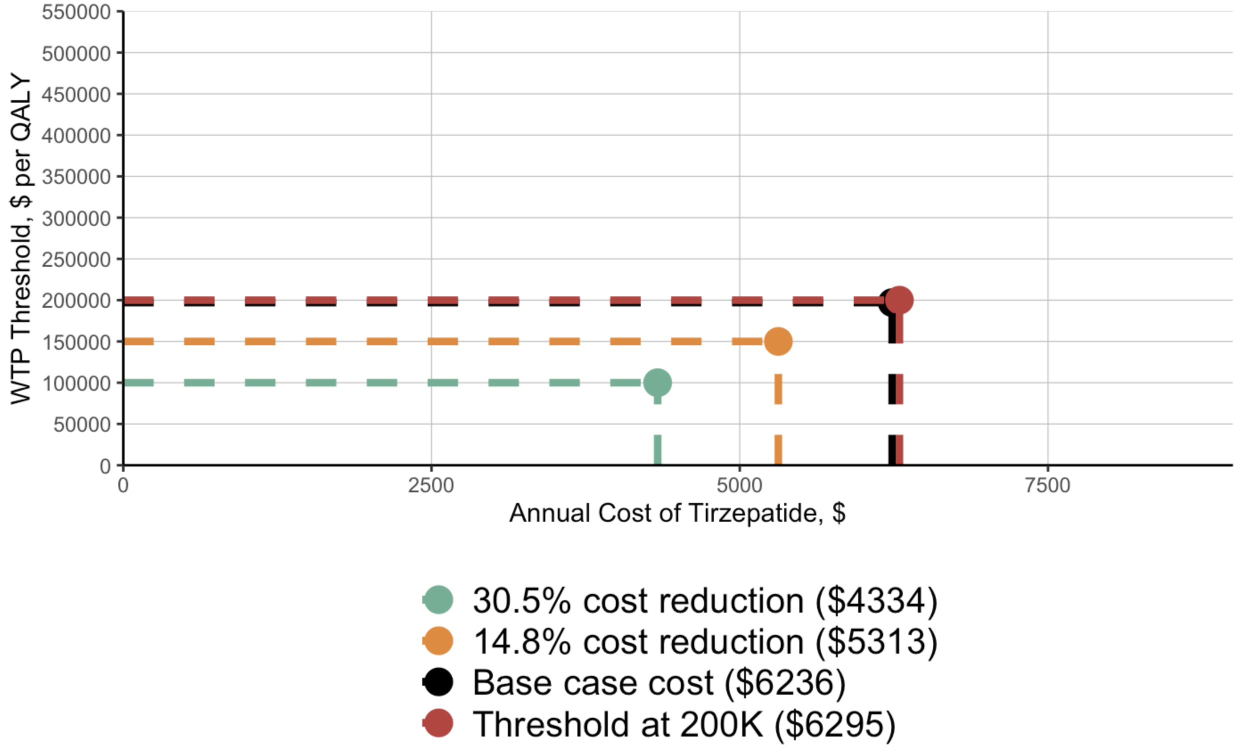

© 2025 Hwang JH et al. *JAMA Health Forum.*

B. Semaglutide

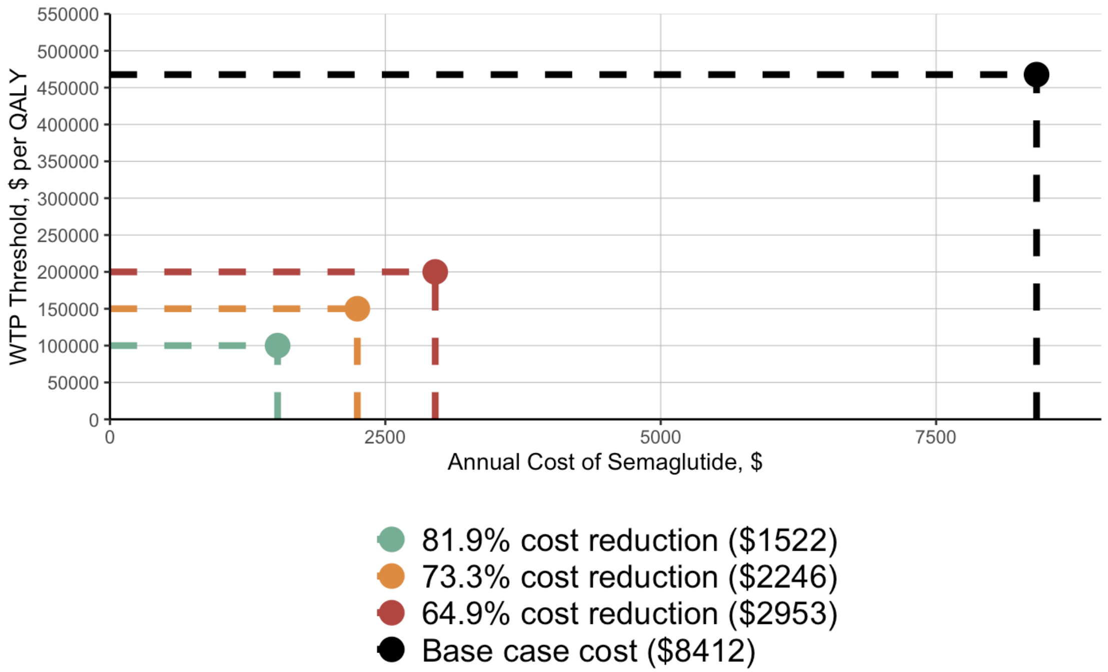

The figure presents a threshold analysis of three anti-obesity medications: tirzepatide (Figure A), and semaglutide (Figure B)

The figure illustrates the required percentage discount on the annual cost of anti-obesity medications (AOMs) to consider it a cost-effective treatment option, using various willingness-to-pay (WTP) thresholds per quality-adjusted life year (QALY) gained. The black dashed line represents the relationship between the base case AOM cost and its corresponding incremental cost-effectiveness ratio (ICER). The green, yellow, and red dashed lines denote the ICER thresholds of $100000, $150000, and $200000 per QALY, respectively. The graph indicates that as the discount rate increases, the ICER decreases, crossing each WTP threshold at specific points. These intersections determine the discounts at which an AOM becomes a cost-effective option compared to lifestyle modification. The analysis is conducted over a lifetime horizon, as per the annual cycle length used in the model.

© 2025 Hwang JH et al. *JAMA Health Forum.*

eReferences

1. *R: A language and environment for statistical computing*. Version 4.2.0. R Foundation for Statistical Computing; 2021. https://www.R-project.org

2. Wilson PWF, Meigs JB, Sullivan L, Fox CS, Nathan DM, D'Agostino RB, Sr. Prediction of Incident Diabetes Mellitus in Middle-aged Adults: The Framingham Offspring Study. *Archives of Internal Medicine*. 2007;167(10):1068-1074. doi:10.1001/archinte.167.10.1068

3. Goff DC, Lloyd-Jones DM, Bennett G, et al. 2013 ACC/AHA Guideline on the Assessment of Cardiovascular Risk. *Circulation*. 2014;129(25_suppl_2):S49-S73. doi:doi:10.1161/01.cir.0000437741.48606.98

4. D'Agostino RB, Russell MW, Huse DM, et al. Primary and subsequent coronary risk appraisal: New results from the Framingham study. *American Heart Journal*. 2000/02/01/ 2000;139(2, Part 1):272-281. doi:https://doi.org/10.1016/S0002-8703(00)90236-9

5. Centers for Disease Control and Prevention: Underlying Cause of Death. 2018;

6. Greenway FL, Fujioka K, Plodkowski RA, et al. Effect of naltrexone plus bupropion on weight loss in overweight and obese adults (COR-I): a multicentre, randomised, double-blind, placebo-controlled, phase 3 trial. *Lancet*. Aug 21 2010;376(9741):595-605. doi:10.1016/s0140-6736(10)60888-4

7. Gadde KM, Allison DB, Ryan DH, et al. Effects of low-dose, controlled-release, phentermine plus topiramate combination on weight and associated comorbidities in overweight and obese adults (CONQUER): a randomised, placebo-controlled, phase 3 trial. *The Lancet*. 2011;377(9774):1341-1352.

8. Jastreboff AM, Aronne LJ, Ahmad NN, et al. Tirzepatide Once Weekly for the Treatment of Obesity. *New England Journal of Medicine*. 2022/07/21 2022;387(3):205-216. doi:10.1056/NEJMoa2206038

9. Wilding JPH, Batterham RL, Calanna S, et al. Once-Weekly Semaglutide in Adults with Overweight or Obesity. *New England Journal of Medicine*. 2021;384(11):989-1002. doi:10.1056/NEJMoa2032183

10. Weintraub MA, D'Angelo D, Tchang BG, et al. Five-year Weight Loss Maintenance With Obesity Pharmacotherapy. *The Journal of Clinical Endocrinology & Metabolism*. 2023;108(9):e832-e841. doi:10.1210/clinem/dgad100

11. Garvey WT, Frias JP, Jastreboff AM, et al. Tirzepatide once weekly for the treatment of obesity in people with type 2 diabetes (SURMOUNT-2): a double-blind, randomised, multicentre, placebo-controlled, phase 3 trial. *The Lancet*. 2023;402(10402):613-626. doi:10.1016/S0140-6736(23)01200-X

12. Hollander P, Gupta AK, Plodkowski R, et al. Effects of Naltrexone Sustained- Release/Bupropion Sustained-Release Combination Therapy on Body Weight and Glycemic Parameters in Overweight and Obese Patients With Type 2 Diabetes. *Diabetes Care*. 2013;36(12):4022-4029. doi:10.2337/dc13-0234

© 2025 Hwang JH et al. *JAMA Health Forum.*

13. Davies M, Færch L, Jeppesen OK, et al. Semaglutide 2.4 mg once a week in adults with overweight or obesity, and type 2 diabetes (STEP 2): a randomised, double-blind, double-dummy, placebo-controlled, phase 3 trial. *The Lancet*. 2021;397(10278):971-984. doi:10.1016/S0140-6736(21)00213-0
14. Garvey WT, Ryan DH, Bohannon NJV, et al. Weight-Loss Therapy in Type 2 Diabetes: Effects of Phentermine and Topiramate Extended Release. *Diabetes Care*. 2014;37(12):3309-3316. doi:10.2337/dc14-0930
15. Garvey WT, Ryan DH, Look M, et al. Two-year sustained weight loss and metabolic benefits with controlled-release phentermine/topiramate in obese and overweight adults (SEQUEL): a randomized, placebo-controlled, phase 3 extension study. *The American Journal of Clinical Nutrition*. 2012/02/01/ 2012;95(2):297-308. doi:https://doi.org/10.3945/ajcn.111.024927
16. Wilding JPH, Batterham RL, Davies M, et al. Weight regain and cardiometabolic effects after withdrawal of semaglutide: The STEP 1 trial extension. *Diabetes, Obesity and Metabolism*. 2022/08/01 2022;24(8):1553-1564. doi:https://doi.org/10.1111/dom.14725
17. Garvey WT, Batterham RL, Bhatta M, et al. Two-year effects of semaglutide in adults with overweight or obesity: the STEP 5 trial. *Nature Medicine*. 2022/10/01 2022;28(10):2083-2091. doi:10.1038/s41591-022-02026-4
18. le Roux CW, Fils-Aimé N, Camacho F, Gould E, Barakat M. The relationship between early weight loss and weight loss maintenance with naltrexone-bupropion therapy. *eClinicalMedicine*. 2022;49doi:10.1016/j.eclinm.2022.101436
19. Aronne LJ, Sattar N, Horn DB, et al. Continued Treatment With Tirzepatide for Maintenance of Weight Reduction in Adults With Obesity: The SURMOUNT-4 Randomized Clinical Trial. *JAMA*. 2024;331(1):38-48. doi:10.1001/jama.2023.24945
20. Lubetkin EI, Jia H, Franks P, Gold MR. Relationship among sociodemographic factors, clinical conditions, and health-related quality of life: examining the EQ-5D in the U.S. general population. *Qual Life Res*. Dec 2005;14(10):2187-96. doi:10.1007/s11136-005-8028-5
21. Kim DD, Wang L, Lauren BN, et al. Development and Validation of the US Diabetes, Obesity, Cardiovascular Disease Microsimulation (DOC-M) Model: Health Disparity and Economic Impact Model. *Med Decis Making*. Oct 16 2023:272989x231196916. doi:10.1177/0272989x231196916
22. Domitrz I, Golicki D. Health-Related Quality of Life in Migraine: EQ-5D-5L-Based Study in Routine Clinical Practice. *Journal of Clinical Medicine*. 2022;11(23). doi:10.3390/jcm11236925
23. Espen Saxhaug K, Knut S, Christofer L, Michael Bjørn R. Impact of chronic headache on workdays, unemployment and disutility in the general population. *Journal of Epidemiology and Community Health*. 2019;73(4):360. doi:10.1136/jech-2018-211127
24. Matza LS, Boye KS, Yurgin N, et al. Utilities and disutilities for type 2 diabetes treatment-related attributes. *Quality of Life Research*. 2007/09/01 2007;16(7):1251-1265. doi:10.1007/s11136-007-9226-0

© 2025 Hwang JH et al. *JAMA Health Forum.*

25. Matza LS, Deger KA, Vo P, Maniyar F, Goadsby PJ. Health state utilities associated with attributes of migraine preventive treatments based on patient and general population preferences. *Quality of Life Research*. 2019/09/01 2019;28(9):2359-2372. doi:10.1007/s11136-019-02163-3
26. Pollack MF, Purayidathil FW, Bolge SC, Williams SA. Patient-reported tolerability issues with oral antidiabetic agents: Associations with adherence; treatment satisfaction and health-related quality of life. *Diabetes Research and Clinical Practice*. 2010;87(2):204-210. doi:10.1016/j.diabres.2009.11.023
27. Gregg E, Jakicic J, Blackburn G, Bloomquist P, Bray G. Look AHEAD Research Group. Association of the magnitude of weight loss and changes in physical fitness with long-term cardiovascular disease outcomes in overweight or obese people with type 2 diabetes: a post-hoc analysis of the Look AHEAD randomised clinical trial. *Lancet Diabetes Endocrinol*. 2016;4:913-921.
28. Zhang P, Atkinson KM, Bray GA, et al. Within-Trial Cost-Effectiveness of a Structured Lifestyle Intervention in Adults With Overweight/Obesity and Type 2 Diabetes: Results From the Action for Health in Diabetes (Look AHEAD) Study. *Diabetes Care*. 2020;44(1):67-74. doi:10.2337/dc20-0358
29. VA Federal Supply Schedule Service. Accessed December 10, 2023. https://www.fss.va.gov/
30. Ippolito BN, Levy JF. Estimating the Cost of New Treatments for Diabetes and Obesity. *AEI Economic Perspectives*. American Enterprise Institute; 2023. https://www.aei.org/wp-content/uploads/2023/09/Estimating-the-Cost-of-New-Treatments-for-Diabetes-and-Obesity.pdf
31. Hernandez I, Sullivan SD. Net prices of new antiobesity medications. *Obesity*. 2024/01/16 2024;NA(NA)doi:https://doi.org/10.1002/oby.23973
32. Nelson S, Whitsel L, Khavjou O, Phelps D, Leib A. Projections of cardiovascular disease prevalence and costs. *RTI International*. 2016;
33. Cawley J, Biener A, Meyerhoefer C, et al. Job Absenteeism Costs of Obesity in the United States: National and State-Level Estimates. *Journal of Occupational and Environmental Medicine*. 2021;63(7)
34. Park J, Bigman E, Zhang P. Productivity Loss and Medical Costs Associated With Type 2 Diabetes Among Employees Aged 18–64 Years With Large Employer-Sponsored Insurance. *Diabetes Care*. 2022;45(11):2553-2560. doi:10.2337/dc22-0445
35. Using Appropriate Price Indices for Expenditure Comparisons. Medical Expenditure Panel Survey. Accessed February 15, 2024, https://meps.ahrq.gov/about_meps/Price_Index.shtml
36. Dakin HA, Leal J, Briggs A, Clarke P, Holman RR, Gray A. Accurately Reflecting Uncertainty When Using Patient-Level Simulation Models to Extrapolate Clinical Trial Data. *Med Decis Making*. May 2020;40(4):460-473. doi:10.1177/0272989x20916442
37. Rubin DB. *Multiple Imputation for Nonresponse in Surveys*. Wiley-Interscience; 2004.
38. *Cost-Effectiveness in Health and Medicine*. Oxford University Press; 2016. https://doi.org/10.1093/acprof:oso/9780190492939.001.0001

© 2025 Hwang JH et al. *JAMA Health Forum.*

39. Weiss T, Carr RD, Pal S, et al. Real-World Adherence and Discontinuation of Glucagon-Like Peptide-1 Receptor Agonists Therapy in Type 2 Diabetes Mellitus Patients in the United States. *Patient Prefer Adherence*. 2020;14:2337-2345. doi:10.2147/ppa.S277676
40. Sikirica MV, Martin AA, Wood R, Leith A, Piercy J, Higgins V. Reasons for discontinuation of GLP1 receptor agonists: data from a real-world cross-sectional survey of physicians and their patients with type 2 diabetes. *Diabetes Metab Syndr Obes*. 2017;10:403-412. doi:10.2147/dmso.S141235
41. Davies EW, Matza LS, Worth G, et al. Health state utilities associated with major clinical events in the context of secondary hyperparathyroidism and chronic kidney disease requiring dialysis. *Health and Quality of Life Outcomes*. 2015/06/30 2015;13(1):90. doi:10.1186/s12955-015-0266-9
42. Benjamin EJ, Virani SS, Callaway CW, et al. Heart Disease and Stroke Statistics-2018 Update: A Report From the American Heart Association. *Circulation*. Mar 20 2018;137(12):e67-e492. doi:10.1161/CIR.0000000000000558
43. Centers for Disease Control and Prevention. Underlying Cause of Death 1999-2016. Accessed September 21, 2018. https://wonder.cdc.gov/wonder/help/ucd.html#
44. Pharmaceutical Pricing Data. The Federal Supply Schedule (FSS) Service. https://www.va.gov/opal/nac/fss/pharmprices.asp
45. U.S. Centers for Medicare & Medicaid Services. Medicare Provider Utilization and Payment Data: Inpatient. 2019. https://www.cms.gov/Research-Statistics-Data-and-Systems/Statistics-Trends-and-Reports/Medicare-Provider-Charge-Data/Inpatient2017.html

© 2025 Hwang JH et al. *JAMA Health Forum.*
