# Supplementary material for: Lifetime Health Effects and Cost-Effectiveness of Tirzepatide and Semaglutide in US Adults
Source: JAMA Health Forum. 2025 Mar 14;6(3):e245586. doi: 10.1001/jamahealthforum.2024.5586 (PMC11909610; doi:10.1001/jamahealthforum.2024.5586)
Supplement: Supplement 2. — Data sharing statement [file jamahealthforum-e245586-s002.pdf]

Data Sharing Statement

Hwang. Lifetime Health Effects and Cost-Effectiveness of Tirzepatide and Semaglutide in US Adults. *JAMA Health Forum*. Published March 14, 2025.
doi:10.1001/jamahealthforum.2024.5586

Data
Data available: No

Additional Information
Explanation for why data not available: All data utilized in the model are sourced from published literature and publicly accessible databases, all of which are cited in our manuscript.
